# Can we do better in identifying child developmental conditions early? Sensitivity and specificity of the Watch Me Grow Integrated (WMG-I) digital developmental screening and surveillance tool in Australia

**DOI:** 10.1136/bmjopen-2026-117341

**Published:** 2026-09-25

**Authors:** Christa Lam-Cassettari, Kate Short, Pankaj Garg, Joseph Descallar, Feroza Khan, Catherine Bradford, Isabella Andersen, Bin Jalaludin, Siaw-Teng Liaw, Susan Woolfenden, Aaron Chambers, Margo Pritchard, Raghu Lingam, K D Lawson, Andrew Page, Jane Kohlhoff, Catherine Marron, Helen Heussler, Dana Newcomb, Nguyet de Mello, Elisabeth Murphy, Paul B Colditz, Micheal Kundukulam, Dimuthu Samaranayake, Shanti Raman, Faye Southcombe, The Watch Me Grow Study Team, James Scott, Valsamma Eapen

**Affiliations:** 1 School of Clinical Medicine, Discipline of Psychiatry & Mental Health, University of New South Wales, Sydney, New South Wales, Australia; 2 Ingham Institute for Applied Medical Research, Liverpool, New South Wales, Australia; 3 Infant, Child and Adolescent Mental Health Service, South Western Sydney Local Health District, Liverpool, New South Wales, Australia; 4 Speech Pathology, South Western Sydney Local Health District, Liverpool, New South Wales, Australia; 5 School of Health Sciences, The University of Sydney, Sydney, New South Wales, Australia; 6 School of Clinical Medicine, University of New South Wales, Sydney, New South Wales, Australia; 7 Academic Unit of Infant, Child and Adolescent Psychiatry, South Western Sydney Local Health District, Liverpool, New South Wales, Australia; 8 Ingham Institute, Liverpool, New South Wales, Australia; 9 Child Health Research Centre, The University of Queensland, Brisbane, Queensland, Australia; 10 South Western Sydney Local Health District, Liverpool, New South Wales, Australia; 11 School of Public Health and Community Medicine, UNSW Australia, Fairfield, New South Wales, Australia; 12 Community Paediatrics, Sydney Local Health District, Camperdown, New South Wales, Australia; 13 Central Clinical School, Sydney Medical School, Faculty of Medicine and Health, The University of Sydney, Sydney, New South Wales, Australia; 14 Integrated Care, Children’s Health Queensland, South Brisbane, Queensland, Australia; 15 Child Health Research Centre Level 6, The University of Queensland, Brisbane, Queensland, Australia; 16 School of Clinical Medicine, Population Child Health Research, University of New South Wales, Sydney, New South Wales, Australia; 17 Western Sydney University, Penrith, New South Wales, Australia; 18 Translational Health Research Institute, Western Sydney University, Penrith, New South Wales, Australia; 19 Queensland Child & Youth Clinical Network, Brisbane, Queensland, Australia; 20 Children’s Health Queensland Hospital and Health Service, South Brisbane, Queensland, Australia; 21 Child Development, Queensland Children’s Hospital, Brisbane, Queensland, Australia; 22 Centre fro Children’s Health Research, University of Queensland, Brisbane, Queensland, Australia; 23 The University of Queensland Primary Care Clinical Unit, Herston, Queensland, Australia; 24 NSW Ministry of Health Population and Public Health Division, St Leonards, New South Wales, Australia; 25 UQCCR, University of Queensland, Brisbane, Queensland, Australia; 26 The Sydney Children’s Hospitals Network, Westmead, New South Wales, Australia; 27 University of New South Wales School of Women's and Children’s Health, Randwick, New South Wales, Australia; 28 Community Paediatrics, South Western Sydney Local Health District, Liverpool, New South Wales, Australia; 29 University of New South Wales, Sydney, New South Wales, Australia; 30 The University of Queensland, Brisbane, Queensland, Australia; 31 University of Queensland Centre for Clinical Research, Herston, Queensland, Australia; 32 Child and Youth Mental Health Service (CYMHS), Children’s Health Queensland, Herston, Queensland, Australia

**Keywords:** General Practice, Community child health, Child, Developmental neurology & neurodisability

## Abstract

**Objectives:**

While child developmental checks are universally recommended, engagement with developmental surveillance programmes is low globally. To address this policy to practice gap, this study examined the diagnostic accuracy of the ‘Watch Me Grow’ Integrated (WMG-I) digital developmental screening and ongoing surveillance approach.

**Design:**

This sub-study examined diagnostic accuracy of the WMG-I digital approach compared with routine assessment (service as usual (SaU)) in a prospective sample.

**Participants, setting and intervention:**

Parents of 16-to-35-month-old children attending 40 general practices (randomised to WMG-I or SaU) in culturally diverse metropolitan New South Wales and Queensland, Australia, participating in a longitudinal cluster randomised controlled trial. Parents in the WMG-I group completed the WMG-I digital weblink, including a primary screener and automated secondary screener (if concerns were reported). On completion, results and recommendations were transmitted to the parent and general practitioner (GP) allowing discussion and further assessment. This was compared with SaU GP assessment. Once engaged, based on child’s date of birth, WMG-I sends automated reminders to take the check again at the next recommended ages, thereby allowing ongoing developmental monitoring/surveillance.

**Primary outcome measures:**

All children with identified concerns and a 10% systematic sample without concerns received a reference standard assessment using the Mullen Scale of Early Learning, Autism Diagnostic Observation Schedule and Vineland Adaptive Behaviour Scale. The primary outcome was diagnostic accuracy of WMG-I compared with SAU on the reference standard assessments.

**Results:**

Of the 655 children in the study at age 2 years, 151 children received reference standard assessment. There was a statistically significant difference in the diagnostic accuracy, with 93.9% sensitivity in the WMG-I group (95% CI 88.1% to 99.7%) and 75.9% (95% CI 57.2% to 94%) in the SaU group (p=0.035); specificity was 71.4% (95% CI 54.7% to 88.1%) and 66.7% (95% CI 44.9% to 88.4%) (p=0.743), respectively.

**Conclusions:**

Universal digital surveillance programmes such as WMG-I can enhance time and service efficiency to accurately identify developmental needs. Leveraging routine primary care contact such as immunisation visits to administer WMG-I digital offers a scalable solution for increasing the uptake of developmental surveillance at a population level internationally.

**Trial registration number:**

ANZCTR12621000680864.

**STRENGTHS AND LIMITATIONS OF THE STUDY**

- The Watch Me Grow (WMG) programme is novel in empowering parents to engage in digital developmental checks leveraging opportunistic primary care visits.
- The WMG weblink for developmental screening and automated reminders for ongoing surveillance demonstrates a sensitive and scalable solution for early identification of developmental concerns.
- Leveraging childhood immunisation and other routine service contacts with primary care practitioners offer equitable reach to all children.
- Study participants were recruited from a group of general practitioners that expressed interest in developmental screening and surveillance and general practitioners who agreed to participate in the study, which may limit the generalisability of the results.

## Introduction

 Delay in early identification of developmental issues especially in disadvantaged populations has significant adverse flow-on effects due to missing early intervention opportunities and consequent long-term disease burden.^1^ Hence, early identification via universal developmental screening and ongoing surveillance in the preschool years is recommended best practice.^2^ In this regard, the Australian Early Years Strategy^3^ supporting the first 2000 days from pregnancy to start of school is a national policy and research priority to ensure all children can flourish. However, up to one in four Australian children are ‘developmentally vulnerable’ at the start of school,^4^ with rates as high as one in two to three among disadvantaged populations. Recent data estimate that the annual cost of late intervention is $22 billion.^5^ Despite significant investment in early child services through state and national programmes, engagement with early developmental checks remains low, with only 20–30% uptake at 12–18 months in some jurisdictions.^6^ Further, access to early identification and timely support for developmental issues is hindered by the complex nature of the Australian child and family service system with fragmentation and service delivery gaps.^7^

Research has shown that currently the greatest obstacle to children receiving early identification and support is an *inverse care law* whereby children from the most disadvantaged backgrounds with the greatest developmental risk are least likely to engage with services early.^7 8^ Qualitative data indicate that facilitators to developmental surveillance uptake include accessibility, continuity of care, visits being combined with another mandatory service (eg, immunisation) and for multicultural families, language concordance with the service provider.^9^ While a high proportion of children access the federally funded general practice services for health-related needs including immunisation during the preschool years, they miss out on accessing the state-based developmental health services including developmental checks.

Thus, there is a significant gap in policy to practice resulting in missed opportunities for early identification and intervention. Addressing this inequity requires a fundamental shift to reaching children who are currently not engaging with routine health services by leveraging opportunistic service contacts and providing supports using a proportionate universalism framework—universal services plus targeted supports commensurate with needs.^10^

One such opportunity is leveraging childhood immunisation visits to engage and empower parents to complete child developmental checks with 87–89% of Australian children receiving immunisation at 12 and 24 months of age.^11–13^ The opportunistic use of ‘Watch Me Grow’, a digital (web-based) developmental surveillance platform (with translations available), has been found to be feasible and acceptable among families from culturally and linguistically diverse, socioeconomically disadvantaged and regional/rural communities.^14^ With preliminary evidence for its effectiveness^15^ in general practice and community settings, this has the potential to decrease the current inequity in early identification of developmental issues including autism.^16 17^ The current study is part of a prospective longitudinal cluster-randomised controlled trial evaluating the digital ‘Watch Me Grow’ Integrated (WMG-I) programme compared with routine surveillance as usual (SaU).^18^ The specific aim of this sub-study was to test the diagnostic accuracy for identifying any developmental condition in WMG-I compared with SaU.

## Methods

### Patient and public involvement

The acceptability and utility of the WMG-I weblink were developed and assessed with parents and health practitioners in a previous study.^19^

### Study design

The data in this article were collected as part of a prospective longitudinal cluster randomised controlled trial to evaluate the digital ‘Watch Me Grow’ developmental screening and ongoing monitoring programme for children from 18 to 48 months (the WMG-I study; for further information, see the study protocol^18^ and online supplemental file 1).

### Randomisation and masking

40 general practices were recruited and randomised to either implement the digital WMG-I approach for developmental surveillance or to routine care, SaU in South-West Sydney, New South Wales (NSW) and Brisbane, Queensland (QLD), with 20 practices randomised to the WMG-I group and 20 to the SaU group. Randomisation was conducted using minimisation across two factors, state and general practitioner (GP) size,^18^ by an independent statistician in the statistical software R using the ‘Minirand’ package. Members of the research team, GPs nor participants had any influence on randomisation.

### Participants

All parents were invited to participate in the WMG-I study when presenting with a child aged 16–35 months at participating general practices for immunisation or other healthcare needs between 2023 and 2025. Study locations reflect areas of large healthcare service provision with populations>1 million people, with approximately 40% of residents born overseas.^20^ The socio-demographic characteristics of the study locations, including proportion of male births,^21^ average birth weight, speaking languages in addition to English at home and holding a post school qualification, align with the Australian census data for South Western Sydney and Brisbane metropolitan areas.^20–22^

### Procedures

After informed consent, all parents completed sociodemographic questions as part of the trial entry. Following this, a parent/child attending a WMG-I practice completed the WMG-I weblink (while in the waiting room or prior to the appointment) to ascertain the developmental progress of the child. Translations in Arabic, Vietnamese and Simplified Chinese language were made available as these are the most commonly used languages in the study sites.^20^ The general developmental surveillance tools that were used in the WMG-I weblink were the same as those recommended by the respective state health departments (ie, Learn the Signs Act Early in New South Wales^23^ or Parent Evaluation of Developmental Status in Queensland^24^), and the 10-item Quantitative Checklist of Autism in Toddlers was also included.^25^ If the parent reported any concerns in their responses to the items in the primary screening questionnaires, the embedded algorithm automatically guided the parent to take the Ages and Stages Questionnaire third edition^26^ as a secondary screening tool in both the NSW and QLD cohorts. Based on the overall results, automated feedback was provided on the screening outcome to the parent along with anticipatory guidance and resources pertaining to developmental milestones for a child of that age. The screening results with automated scoring and any recommendations (as applicable) were transmitted instantaneously to the GP using the Health Link secure electronic messaging system to ensure they could be integrated into the patient files stored in the practice management software (eg, Best Practice, Medical Objects) used by the general practice. This allowed the parent and the GP to discuss the results and management options where any concerns were identified then and there as part of the regular consultation. Based on the date of birth, WMG-I platform sends automated reminders to take the checks again at the next recommended ages, thereby allowing ongoing developmental monitoring or surveillance.

A parent/child attending a SaU group general practice received routine care by their GP including clinical judgement of any parent concern and/or administration of any screeners the GP deemed relevant to their service provision. For each SaU participant, the GP completed a four-item case report online, noting if any developmental screening was done, whether any screening tools were used, if any developmental needs were identified and whether any applicable referrals were made for further investigation and management.

Total scores were calculated for each screening tool used in the WMG-I weblink with automated scoring used to ascertain whether the children screened positive or negative in their developmental assessment. Following this, all children who screened positive for probable developmental disability from WMG-I or GP report of probable developmental disability for SaU participants; as well as a systematic sample of 10% (every 10th participant was contacted) who screened negative (on the WMG-I weblink or were reported to have no concerns by the SaU GP) from the baseline cohort were invited to participate in a reference standard developmental assessment when aged ≥24 months of age. The children were assessed by a researcher trained in the use of these reference standard developmental assessment tools and who was blind to the cluster status and the screening results of the children being assessed (for details of the protocol, see^18^). The diagnostic outcome was based on the reference-standard assessments using three assessment measures: the Mullen Scale of Early Learning (MSEL),^27^ Vineland Adaptive Behaviour Scales Third Edition (Vineland-3)^28^ and the Autism Diagnostic Observation Schedule (ADOS; the child’s age and verbal ability were used to determine whether the Toddler Module, ADOS-1 or ADOS-2 was administered).^29^ All standard reference assessments require stringent training in reliable administration and provide norm-based standardised scores to determine the presence of any developmental risk compared with same aged peers. Additionally, a clinical advisory group oversaw all assessment interpretations in case review meetings.

### Outcomes

A positive diagnostic outcome for the standard reference assessment was determined when a child met diagnostic criteria on the (1) ADOS (indicating probable autism spectrum disorder (ASD)) or (2) scored below the 10th percentile on any domain of both MSEL and Vineland-3 (indicating probable developmental delay (DD) or (3) met diagnostic criteria on ADOS and scored below the 10th percentile on any domain of both MSEL and Vineland-3 (indicating probable ASD+DD).

As illustrated in figure 1, the patient flow through the various screening outcomes and reference-standard assessment outcomes were used to calculate the sensitivity and specificity of the WMG-I and the SaU approach. The STARD reporting guideline was used to draft this article.^30^

**Figure 1:**
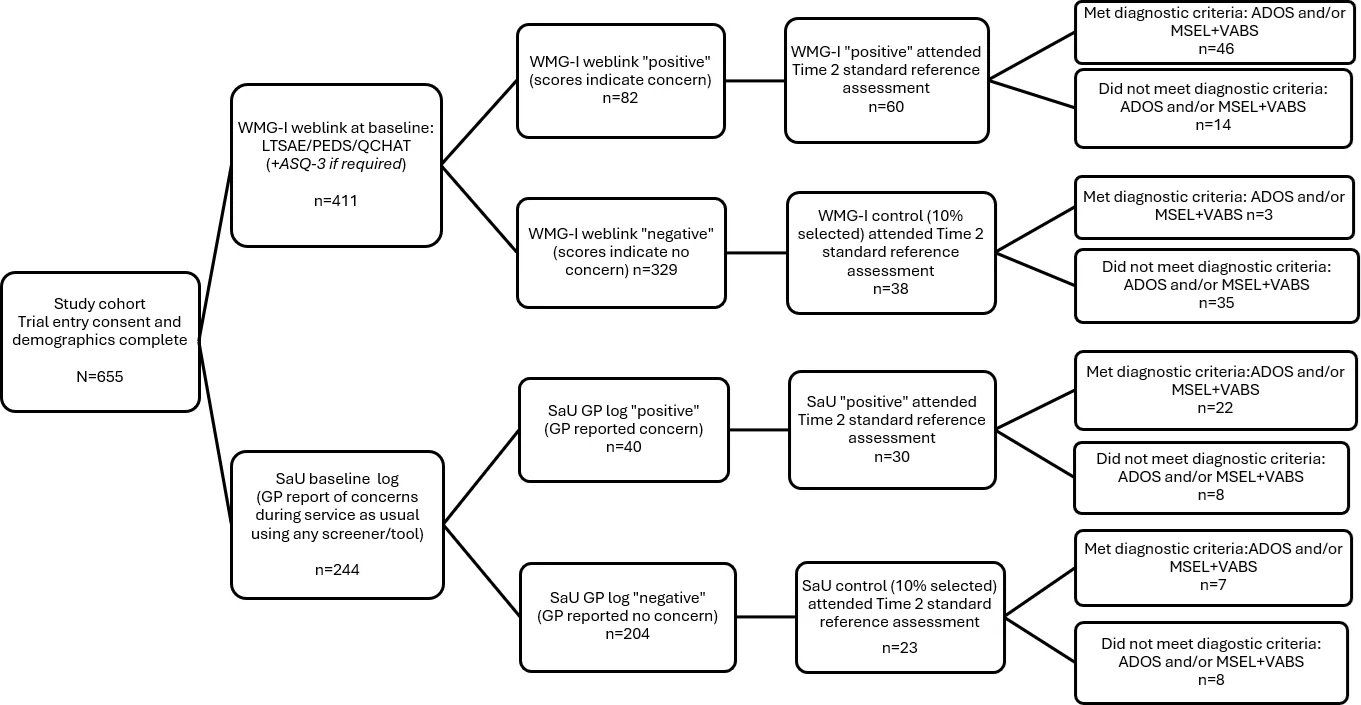
Study cohort and patient flow through the baseline test for the Intervention group (WMG-I weblink) and SaU group (GP log of any concern) and Time 2 standard reference assessment (MSEL, VABS, ADOS). Time 2 outcome of ‘met diagnostic criteria’ reflects cases where ADOS diagnostic criteria were met, or scores were <10th percentile on both MSEL and VABS. ADOS, Autism Diagnostic Observation Schedule; ASQ-3, Ages & Stages Questionnaire version 3; GP, general practitioner; LTSAE, Learn the Signs Act Early; MSEL, Mullen Scale of Early Learning; PEDS, Parents Evaluation of Developmental Status; QCHAT-10, Quantitative Checklist for Autism in Toddlers 10-Question; SaU, Service as Usual; VABS, Vineland Adaptive Behaviour Scales; WMG-I, Watch Me Grow Integrated.

### Statistical analysis

Sample size was based on a convenience sample as determined by those who completed the reference standard assessment. Fisher’s exact test was used to analyse baseline socio-demographic characteristics between the two groups of parents and children who attended a standard reference assessment at age 2 years. Sensitivity, specificity and positive and negative likelihood ratios were calculated for the outcomes from the baseline screening assessments compared with outcomes from the reference standard assessments (ADOS, MSEL and Vineland). CIs for these statistics were calculated with a variance inflation factor^31^ to account for clustering by GP. Generalised estimating equation with logit link and binomial distribution was used to compare the sensitivity and specificity between the groups, clustering by GP. The variance was adjusted to account for the small number of GPs (<40) that were included in this analysis. Area under the curve (AUC) was calculated for the WMG-I and SaU groups, and compared with the Cluster-Robust Obuchowsli method to account for clustering by GP. There were no missing data.

## 
Results

The socio-demographic characteristics of the children who received the reference standard assessment are shown in table 1. Significantly more male than female children attended the assessment in the SaU group (75%) compared with the WMG-I group (54.6%). Of the total of 665 children recruited at baseline, there were no other significant differences between socio-demographic variables for the WMG-I and the SaU groups. At baseline screening, 82 out of 411 (20%) children in the WMG-I group were positive for probable developmental concerns on the WMG-I weblink compared with 40 out of 204 (19.6%) children reported by the SaU GPs as positive for probable developmental disability (p=1). Of these 122 children with probable developmental disability, 90 children and a further 61 children (the systematic sample of 10%) who screened negative participated in the reference standard assessments.

**Table 1:** Socio-demographic characteristics of children who received the reference standard assessment

	WMG-I, n (%)	SaU, n (%)	Total, n	P value
*Sex assigned at birth*				0.035
Male	54 (55.1)	39 (73.6)	93	
Female	44 (44.9)	14 (26.4)	58	
*‘Were there any birth complications’?*				0.067
Yes	16 (16.3)	15 (28.3)	31	
No	77 (78.6)	38 (71.7)	115	
Don’t know	5 (5.1)	0 (0)	5	
Child birth weight, mean (SD)	3.65 (2.03)	3.83 (1.94)		0.6089
*Child born premature*				1
Yes	9 (9.2)	4 (7.6)	13	
No	89 (90.8)	49 (92.5)	138	
*‘Estimated family income’*				0.3015
<$50 000 per year	24 (25)	6 (11.3)	30	
$50 000–$75 000 per year	10 (10.4)	9 (17)	19	
$76 000–$100 000 per year	12 (12.5)	9 (17)	21	
$101 000–$150 000 per year	28 (29.2)	16 (30.2)	44	
>$150 000	22 (22.9)	13 (24.5)	35	
*‘Does your household income meet your costs?’*				0.9669
Not at all	9 (9.4)	6 (11.3)	15	
Yes, just enough	53 (55.2)	29 (54.7)	82	
Yes, with some left over	26 (27.1)	13 (24.5)	39	
Yes, with a lot left over	8 (8.3)	5 (9.4)	13	
*Mother’s ethnicity*				0.1361
Caucasian	31 (31.6)	25 (47.2)	56	
Other	64 (65.3)	26 (49.1)	90	
Caucasian and other	3 (3.1)	2 (3.8)	5	
*Father’s ethnicity*				0.1977
Caucasian	41 (42.3)	23 (44.2)	64	
Other	55 (56.7)	26 (50)	81	
Caucasian and other	1 (1)	3 (5.8)	4	
*Mother’s education level*				0.5201
Year 10	6 (6.1)	5 (9.4)	11	
Year 12	4 (4.1)	5 (9.4)	9	
TAFE (vocational education provider)/post school qualifications	22 (22.5)	10 (18.9)	32	
University—undergraduate degree	38 (38.8)	16 (30.2)	54	
University—postgraduate degree	28 (28.6)	17 (32.1)	45	
*Father’s education level*				0.0618
Year 10	6 (6.4)	2 (3.9)	8	
Year 12	12 (12.8)	17 (32.7)	29	
TAFE*/other post school qualifications	27 (28.7)	11 (21.2)	38	
University—undergraduate degree	29 (30.9)	11 (21.2)	40	
University—postgraduate degree	20 (21.3)	11 (21.2)	31	

*TAFE (Technical and Further Education, a state vocational education provider).

SaU, service as usual; WMG-I, Watch Me Grow Integrated.

Of the 151 children who completed the reference standard assessments, 60 were positive on the automated dual screening weblink in the WMG-I group and 30 had been identified as positive for having probable developmental disability by their GP in the SAU group. Of those who met diagnostic criteria at the standard reference assessment in the WMG-I group, 1 (2%) met only the ADOS criteria, 14 (23%) met the composite criteria (<10th percentile on both MSEL+Vineland-3) and 27 (45%) met criteria on all three assessments. Of those who met the diagnostic criteria in the SaU group, none met only the ADOS criteria, 6 (20%) met the composite criteria (<10th percentile on both MSEL+Vineland-3) and 12 (40%) met criteria on all assessments. A negative score on the ADOS was associated with a negative outcome (no concerns) on the WMG-I screen (p<0.001). See table 2 for further details.

**Table 2:** Categorisation of the standardised reference assessment outcomes for ASD, or DD, or ASD+DD, or neither ASD nor DD on the MSEL, Vineland Adaptive Behaviour Scales Third Edition and ADOS

Risk status at baseline	ASD without DD (ADOS diagnostic criteria met) (n)	DD without ASD (both MSEL+Vineland-3 scored<10 percentile) (n)	ASD+DD (ADOS diagnostic criteria met+MSEL and Vineland-3 <10th percentile) (n)	Does not meet criteria for ASD or DD (n)
WMG-I weblink score ‘positive’ n=60	1	14	27	18
WMG-I weblink score ‘negative’ (10% selected) n=38	1	1	0	36
SaU GP reported ‘positive’ n=30	0	6	12	12
SaU GP reported ‘negative’ (10% selected) n=23	4	0	3	16

‘Positive’ baseline outcome indicates probable developmental disability; ‘negative’ baseline outcome indicates no/low concern.

ADOS, Autism Diagnostic Observation Schedule; ASD, autism spectrum disorder; DD, developmental delay; GP, general practitioner; MSEL, Mullen Scale of Early Learning; SaU, service as usual; WMG-I, Watch Me Grow Integrated.

Table 3 presents the sensitivity and specificity and likelihood ratio results for the WMG-I and SaU groups. The sensitivity in the WMG-I group was 93.9% (95% CI 88.1% to 99.7%), and 75.9% (95% CI 57.2% to 94.6%) in the SaU group. The 18% difference in sensitivity between the WMG-I and SaU group was significant (p=0.035). Given the higher proportion of male children than females in the SaU compared with the WMG-I group, a post hoc exploratory analysis was conducted which showed that after accounting for sex, the difference in sensitivity was not significant (p=0.061). The specificity in the WMG-I group was 71.4% (54.7% to 88.1%) and 66.7% (44.9% to 88.4%) in the SaU group. The 4.7% difference in specificities was not significantly different (p=0.743) and remained non-significant after adjusting for sex (p=0.816).

**Table 3:** Sensitivity, specificity and likelihood ratio results for the WMG-I and SaU groups

Study arm	Baseline risk status	Gold standard assessment positive	Gold standard assessment negative	Sensitivity (95% CI)	Specificity (95% CI)	Positive likelihood ratio (95% CI)	Negative likelihood ratio (95% CI)
WMG-I Group	Positive	46	14	93.9% (88.1% to 99.7%)	71.4% (54.7% to 88.1%)	3.30 (1.82 to 5.92)	0.09 (0.032 to 0.23)
	Negative	3	35				
SaU Group	Positive	22	8	75.9% (57.2% to 94%)	66.7% (44.9% to 88.4%)	2.28 (1.13 to 4.57)	0.36 (0.16 to 0.84)
	Negative	7	16				

Gold standard assessment indicative of probable developmental disability when diagnostic criteria were met on (1) ADOS (probable ASD), (2) MSEL+Vineland-3 (probable DD) or (3) ADOS+MSEL+Vineland-3 (probable ASD+DD).

ADOS, Autism Diagnostic Observation Schedule; ASD, autism spectrum disorder; DD, developmental delay; MSEL, Mullen Scale of Early Learning; SaU, service as usual; WMG-I, Watch Me Grow Integrated.

A positive likelihood ratio (LR) of 3.3, 95% CI (1.82 to 5.92) and negative LR of 0.09, 95% CI (0.03 to 0.23) was found for the WMG-I group, with a positive LR of 2.28, 95% CI (1.13 to 4.57) and negative LR of 0.36, 95% CI (0.16 to 0.84) for the SaU group. This indicates a positive outcome for probable developmental disability at baseline for both groups (WMG-I and SaU) has a moderate increase in the probability of meeting diagnostic criteria at the standard reference assessment. In the WMG-I group, non-identification of probable developmental disability at baseline has a slight decrease in probability of meeting diagnostic criteria at the standard reference assessment, whereas in the SaU group this effect was moderate. The AUC for WMG-I group was 0.827, and for SaU group was 0.713, and the difference was not statistically significant (p=0.151). Given that there were differences in the proportion of males and females in the SaU group, post hoc analysis was conducted of children that were in the SaU group with a positive reference standard result and found that 82% of males (18/22) and 43% of females (3/7) were screened positive at time 1 (p=0.04).

## Discussion

The Australian National Children’s Mental Health and Wellbeing Strategy^32^ and the Productivity Commission Inquiry Report on Mental Health^33^ have called for early identification and intervention of developmental and behavioural problems to improve the life course trajectory of Australian children. However, currently there is no systematic mechanism to reach all children, and the uptake of developmental checks remains low particularly among those from disadvantaged backgrounds and population groups. Our findings of 93.9% sensitivity for the digital WMG-I screening and ongoing surveillance provide evidence for the feasibility and effectiveness of a digital developmental screening and surveillance programme that can be implemented at scale in the primary care setting. An ideal screening tool is one with a high sensitivity and reasonable specificity as was found in this study, as the specificity can be improved by a two-stage process.^34^ In such a two-stage model of developmental screening and ongoing surveillance, an initial screening tool with high sensitivity is used to identify as many children at risk as possible, followed by a more specific secondary screening tool and/or interview by a primary care professional in order to clarify and confirm the developmental concerns with the parent who completed the initial screener; a method that has been recommended as achieving the optimum outcomes.^35^ In view of the high sensitivity and adequate specificity of the digital WMG-I found in Australian GP settings with parents across sociodemographic and cultural and linguistic characteristics engaging with the programme, this approach could offer a unique opportunity for GPs to play a key role in developmental screening and ongoing surveillance programmes, to complement existing State-run child and family health services.

Harnessing the plasticity of the developing brain to improve developmental trajectory outcomes through early intervention and supports^36^ requires early identification of children with developmental vulnerability. However, currently there is significant inequity in the access to developmental services in the preschool years^37 38^ with consequent delay in early identification and they often present only after complexities and comorbidities have set in. Hence, identification of developmental differences as they emerge particularly from the second year in life needs to be a priority. Our findings indicate that this can be achieved by leveraging opportunistic primary care contact. With the uptake of childhood immunisation at around 90% or higher not only in high-income countries such as Australia but also in low-income and middle-income countries, leveraging this opportunistic immunisation contact could support universal and equitable completion of developmental checks. In this regard, the WMG-I weblink could reach children who need it the most and who otherwise are not currently engaging with universal state health services for developmental checks. Further, once parents have engaged, the WMG platform is programmed to send automated reminders at the next age when it is recommended in the Personal Health Record as per state policies (eg, NSW recommends 2, 6, 12, 18, 24, 36 and 48 months of age). This is aligned to the need for ongoing developmental monitoring (surveillance) during the preschool years rather than one-off developmental screening. The dynamic nature of child milestone development makes conjoint screening and ongoing surveillance more effective^39^ in the preschool years as screening at a single time point may miss new issues that may emerge later, or an earlier developmental concern that may resolve as the brain further develops.

Hence, engaging and empowering families to complete a quick developmental screening tool in the context of a GP service contact, such as childhood immunisation or other routine visits, will go a long way to address the current low uptake of the developmental surveillance programme.^6^

## Limitations

Our study evaluated the diagnostic accuracy of the WMG-I digital screening and surveillance tool compared with SaU in a group of GPs that agreed to participate in the study, and they may be more motivated to conduct developmental checks. However, it is noteworthy that despite all GPs expressing interest in developmental screening and surveillance, the lower sensitivity shown by the SaU group (75.9% identified) compared with the WMG-I group (93.9% identified) may be a result of GPs in the SaU missing cases of probable developmental concern as they were not utilising standardised tools for routine developmental screening and surveillance as part of their usual practice. Indeed, the overall rate of cases that screened positive on WMG-I and were later identified as having probable developmental concerns on the standard reference assessment aligns with typical rates of developmental concerns in the general population as indicated by the Australian Early Development Census.^4^ It should also be noted that statistically fewer females (25%) were identified by SAU GPs compared with the proportion of males (75%) identified, indicating that GPs may be less likely to look for and identify developmental problems in female toddlers. This aligns with evidence for a male predominance in the diagnosis of ASD and greater difficulty diagnosing females with ASD.^40^ A recent population study demonstrated that diagnosis of ASD occurs later for females than males, although differences in the ratio of male to female diagnosis reduce as children reach 10 years of age.^41^ We argue that the WMG-I digital screening and surveillance tool will help GPs to systematically engage in developmental screening and indicate when to refer and follow-up actively instead of taking a ‘watch and wait’ approach. It should be noted that implementation of WMG-I weblink into clinical practice will require further ‘buy-in’ and upskilling of GPs/practice nurses in informing parents of the importance of developmental milestone checks and embed tools such as WMGI as part of their routine healthcare to aid in early identification and provision of appropriate supports if concerns are identified. It should also be noted that open and ongoing dialogue between GPs and parents will be needed in supporting uptake of further diagnostic testing. Using tools such as WMG-I alongside routine immunisation programmes, which have around 90% reach, will further enhance the equity and scalability of developmental screening and surveillance programme internationally including in low and middle income countries.

## Conclusion

Findings from this study offer a feasible opportunity to increase the effectiveness of early child development checks using a combined primary and secondary digital screener and dovetailing with the successful childhood immunisation programme. The recent state and federal policy initiatives for developmental surveillance in Australia, including mental health and well-being check as conducted in this study, represent an effective model for a national approach. A programme such as WMG-I when embedded within immunisation policy has the unique opportunity to ensure equitable access to early identification of developmental issues and matching interventions that support ‘all’ children regardless of their cultural, linguistic, socioeconomic or geographic background. The success of the programme in highly multicultural regions of Australia including the availability of translations to different languages suggests that the programme has the potential for dissemination internationally.

## Supplementary material

10.1136/bmjopen-2026-117341 online supplemental file 1

## Data Availability

All data relevant to the study are included in the article or uploaded as supplementary information.
