# Supplementary material for: Can we do better in identifying child developmental conditions early? Sensitivity and specificity of the Watch Me Grow Integrated (WMG-I) digital developmental screening and surveillance tool in Australia
Source: BMJ Open. 2026 Sep 25;16(9):e117341. doi: 10.1136/bmjopen-2026-117341 (PMC13629963; doi:10.1136/bmjopen-2026-117341)
Supplement: Supplementary file 1 [file bmjopen-16-9-s001.pdf]

“Watch Me Grow Integrated approach - WMG- I”: Changing practice to improve universal child health and developmental surveillance in the primary care setting

Summary

**Study Title**	“Watch Me Grow Integrated approach -WMG- I: Changing practice to improve universal child health and developmental surveillance in the primary care setting
**Aims/Objectives**	The overall purpose of this study is to evaluate the effectiveness and economic cost of the Watch Me Grow – Integrated (WMG-I) online intervention and to explore in-depth stakeholder and community requirements to inform Child Health Developmental Surveillance policy.<br><br>Firstly, in a Cluster Randomised Controlled Trial (c-RCT) we aim to assess if WMG-I, a web-based integrated-service approach to child developmental screening and surveillance, increases the proportion of children receiving scheduled surveillance checks, and improves service use and child outcomes from infancy to school age.<br><br>The primary aim is to assess whether the WMG-I online surveillance approach compared to Surveillance as Usual (SaU) in primary care General Practices:<br><br>1. increase scheduled developmental surveillance completion rates at (a) 18 months of age and (b) from 18 months to 4 years of age?<br><br>2. increase test accuracy for identifying diagnostic global developmental delay and autism at 2 years of age?<br><br>3. increase parent and clinician satisfaction with child surveillance and parent health literacy at 4 years of age?<br><br>4. improve child behavioural outcomes and school readiness before 5 years of age?

	The project will also conduct an exploratory economic analysis to compare cost effectiveness of WMG-I intervention with SaU.<br><br>Secondly, this project includes a qualitative study using focus groups and in-depth interviews to explore parents' and practitioner views around the results of the c-RCT and broader implications of child surveillance and referral pathways to inform policy recommendations and translation to clinical practice.
**Study design**	• A cluster randomised controlled trial (c-RCT) with forty GP Practices (across Brisbane QLD and Sydney NSW) randomised to WMG-I or SaU.<br>• A qualitative study that will involve focus groups and in-depth interviews with parents/caregivers and practitioners/other key stakeholders conducted after completion of the c-RCT to develop practice and policy recommendations.
**Planned sample size**	For the c-RCT, 2000 children will be recruited (1000 WMG-I and 1000 SaU) across 40 GP practices. The qualitative study will involve 6 parent focus groups (up to 10 participants per focus group), 6 professional focus groups, along with in-depth interviews with professionals conducted after the c-RCT.
**Inclusion criteria**	c-RCT: GP practices that offer child immunization and have the capacity to recruit approximately 50 children in one year. Children and their parents/caregivers presenting at participating GP practices for 18 month child immunisation or other health care needs (range: 16 to 35 months).<br><br>Qualitative study: parents/caregivers of children ≤ 5 years of age and clinicians and policy makers involved in any aspect of child developmental surveillance.
**Study procedures**	cRCT

SITES: The c-RCT will be conducted in two sites: 40 GP practices across NSW (South Western Sydney Local Health District: SWSLHD) and Queensland (The University of Queensland) and randomly allocated to two groups: (1) SaU (N=20), or (2) WMG-I (N=20). A total of 2000 children (1000 in each group) will be recruited by GP staff as they present for appointments around 24 months of age (range: 16 to 35 months) over a 12 month period.

TRIAL ENTRY 24 MONTHS: All parents/caregivers will be consented, enrolled and complete baseline and socio-demographic questions online via study provided iPads in the GP waiting rooms prior to their appointment.

INTERVENTIONS: The SaU group will receive child developmental surveillance as it is usually conducted by each GP practice.

The WMG-I group will: (1) complete standardised developmental screens (with automated scoring emailed to the parent and GP) whilst parent is in the waiting room; (2) complete GP-parent consultation based on the screening results where results and management options are discussed, those who screen positive are referred to the research Child and Family Health Nurse who coordinates a 'Triage and Review Team' to recommend, implement and follow-up referral pathways with GPs and parents. From 18 months to 4 years of age automated reminder emails and a text message will be sent to alert parents to the ongoing recommended surveillance screens (those outlined in their child's Personal Health Record). Parents complete these online and steps (1) and (2) above are repeated.

FOLLOW-UP AT 2 YEARS OF AGE: children in both groups who are identified at trial entry by the developmental screening processes to have a developmental concern and a 10% random sample of screen negative cases will be assessed using a battery of 'gold standard'

developmental diagnostic measures by blinded assessors. This data will be used to test the screening accuracy between the two groups.

FOLLOW-UP AT 3 YEARS OF AGE: Parents of WMG-I group will receive an email or text invitation to complete online standardised developmental screens similar to trial entry and both the GP and parents will receive the results. Parents of all children in both groups will complete online questionnaires to capture their service use, referral uptake, surveillance satisfaction, parental health, health literacy and cost questionnaires.

FOLLOW-UP 4 YEARS OF AGE: Parents of WMG-I group will receive an email or text invitation to complete online standardised developmental screens similar to 3 years and both the GP and parents will receive the results. Parents of all children in both groups will complete online questionnaires to capture their service use, referral uptake, surveillance satisfaction parental health, health literacy and cost questionnaires. School readiness will be assessed in all children in both groups by parents who consent for the research team to contact the children’s teachers to access their transition to school statements, and assessed for behavioural outcomes using the Developmental Profile-4 .

THE QUALITATIVE STUDY: 12 focus groups (6 per site). Parents of infant/toddler/pre-school aged children will be recruited by invitations through early childhood education and care settings, community health, general practices and local community groups (1 group per site). In addition, two parent focus groups will be conducted with parents participating in WMG-I (1 group per site) and SaU (1 group per site), recruited from those who completed the 3 year assessments. The professional focus groups will be conducted through partner organisations to include allied health, CFHNs, practice nurses (3 groups per

	site), GPs, intervention service providers, policy makers. Early Childhood Education and Care (ECEC) representatives who are unable to participate in focus groups will be offered in-depth interviews. Up to 20 in-depth interviews (10 per site) will be conducted.
**Analysis considerations**	Randomisation will be stratified by state (NSW, QLD) and GP practice size to ensure equal numbers between groups. The allocation ratio will be 1:1. The randomisation list will be generated centrally by an independent statistician from UNSW.<br><br>Based on our previous work we estimate that by 4 years of age 10% of children will have completed their state-based child developmental screening surveillance schedule. We have postulated that that the WMG-I intervention may improve these rates to 90%.<br><br>Total sample size = 2,000. Sample sizes of 1000 in group one (WMG-I) and 1000 in group two (SaU) were obtained by sampling 20 groups with 50 subjects each in each group, to achieve 100% power to detect a difference between the group proportions of 0.8. The group two proportion is 0.1. The group one proportion is assumed to be 0.1 under the null hypothesis and 0.9 under the alternative hypothesis. The test statistic used is the two-sided Z test (pooled). The significance level of the test was 0.05.
**Study duration**	The project will run for 5 years, from January 2022 to December 2026.

Data Source and Population

<u>At enrolment into the study</u> at 18 (16-35) months, all parents complete the online questions: (a) *child* (DOB, gender, prematurity, birthweight, breastfeeding, complications, country of birth, child health); (b) *parent* (DOB, relationship to child,

gender, country of birth, language spoken, relationship status, social support, contact information); (c) *family* (postcode, income, income meets costs, education, neighbourhood satisfaction, mental health of self/partner, substance use of self/partner, learning problems of self/partner, cigarette use in pregnancy, alcohol use in pregnancy); and (c) *service use* (developmental checks, facility attended and satisfaction at 6 and 12 months).

The WMG-I group will also complete the following:

Three primary screens taking 2-5 minutes each to complete:

- **Learn the Signs Act Early (LTSAE)** [21], is a checklist used to monitor child development in the domains of Social/Emotional, Language/Communication, Cognitive (learning, thinking, problem-solving) and Movement/Physical development milestones and takes a few minutes to complete. Parents use the checklist to monitor if their child is meeting age related milestones and identify when a child requires further developmental screening (75% or more of children can do each milestone). For each instance that a child does "not yet" demonstrate a milestone, a score of 1 is issued and the total score is summed to determine the number of milestones the child is not yet reaching. A score of 1 or more on any of the milestones will be classified a "positive" result and trigger follow up assessment with the secondary screener – ASQ-3.
- **Parent Evaluation of Developmental Status (PEDS)** [19], a 10-item screening questionnaire that asks parents about concerns they have in relation to the following domains of child development: global/cognitive, expressive language and articulation, receptive language, fine and gross motor, behaviour, self-help, socialisation and academic. PEDS uses a scoring system from A through to E, where A is the most predictive of disability. PEDS has been reported against gold standard testing for developmental disability in the general paediatric population where the prevalence of disability was 2.9%, demonstrating a sensitivity of 74–79%, specificity of 70–80%. A PEDS score of path A (multiple significant concerns highly predictive of disability) or path B (B=a single predictive concern) will be classified a "positive" result and trigger follow up assessment with the secondary screener – ASQ-3.
- **Quantitative Checklist for Autism in Toddlers – 10-item (Q-CHAT-10)** [20], is a 10-item "red-flag" screening questionnaire that can be used to determine if a child requires referral for a full diagnostic assessment. The questions ask parents about the frequency of behaviours/symptoms known to be typical in children with autistic disorder and takes about three minutes to complete. The Q-CHAT-10 has sensitivity of 0.91, specificity of 0.89, PPV of 0.58, and internal

consistency of 0.85 (22). A score of ≥3 will be classified a "positive" result and will trigger follow up assessment with the secondary screener – ASQ-3.

Secondary screens for failed primary screens, which take 5-10 minutes to complete

- **Ages and Stages Questionnaire-Third Edition (ASQ-3)** [22] assesses developmental progress in children between the ages of 1 month and 5.5 years. Questions are divided into five areas of development including: Communication, Gross Motor, Fine Motor, Problem Solving and Personal-Social. The *ASQ-3 will only be completed by those who screen positive for developmental risk on PEDS, LTSAE or Q-CHAT.* ASQ-3 has been reported against gold standard testing for developmental disability in more than 18,000 children from the general paediatric population, demonstrating a sensitivity of 85-92%, specificity of 78–92%. Scores that fall below the standard range on any of the five developmental areas will be classified as a "disability outcome".

Parents will also complete the following measure:

- **Kessler Psychological Distress Scale (K6)** [24, 25], a 6 item scale to measure to yield a global measure of distress based on questions about anxiety and depressive symptoms experienced in the past 4 weeks. Responses are summed to give a total score. A cut point of 18 or more will flag the presence of serious mental illness.

We provide English, Arabic, Vietnamese and Chinese language formats of the screening measures.

Parents in the **SaU** group will be assessed by GP in accordance with their standard usual practices and these will be recorded.

At the 2 year assessment, all children in WMG-I and SaU who are identified at developmental risk (at 16-35 months) plus a randomly selected sample of 10% not at risk, will be invited to participate in the 2 year gold standard developmental assessment. For those children who are 24 months at the time of recruitment, the gold standard assessment will be delayed by 2 months to ensure that the parent/child does not receive too many assessments at the one time (especially for those identified at risk

for developmental concerns). The assessor (a clinical psychologist) will be blind to the participant group status and to the results of the surveillance measures at trial entry. The following gold-standard diagnostic based test will be administered by the assessor and compared with age appropriate cut-off scores (see also Table 2):

- **Mullen Scale of Early Learning (MSEL)** [26], a standardised measure of nonverbal and verbal development in children from birth to 68 months of age. Five developmental domains are assessed including: gross motor, fine motor, visual reception, receptive language and expressed language.
- **The Vineland Adaptive Behavior Scales Third Edition (Vineland-3)** [27] is a standardized assessment tool that utilizes semi-structured interview to measure adaptive behavior and support the diagnosis of intellectual and developmental disabilities, autism, and developmental delays.
- **Autism Diagnostic Observation Schedule Toddler Module (ADOS-2)** [28], the only tool that provides a semi-structured direct assessment of the child's social and communication skills and behaviour.

The 2 year assessment will take approximately 1.5 to 2 hours to complete and will be videotaped (with parent consent) to enable coding for inter-rater reliability on measures for a sub-set of the sample.

Scoring Appendix: Risk level for study measures

<table>
<tr><td>Study Timepoint</td><td>Measure</td><td>OUTCOME “at-risk” vs. “low/no concern”</td><td>Pathway</td></tr>
<tr><td>Baseline,
3 years,
4 years</td><td>PEDS (QLD)</td><td>Path-A: ≥2 or more predictive concerns = “at-risk”
Path-B: 1 predictive concern = “at-risk”
0 predictive concerns = “no concern”</td><td rowspan="3">PRIMARY screen positive for “at-risk” triggers a SECONDARY screen with the ASQ-3</td></tr>
<tr><td>Baseline
3 years,
4 years</td><td>LTSAE (NSW)</td><td>Score ≥1 concerns = “at-risk”
Score 0 = “no concern”</td></tr>
<tr><td>Baseline,
3 years.</td><td>Q-CHAT-10</td><td>Score >3 concerns = “at-risk”
Score ≤3 or fewer concerns = “low concern”
Score 0 = “no concern”</td></tr>
<tr><td>Baseline,
3 years,</td><td>ASQ-3</td><td>At 18 months “at-risk” cut-off:
Communication score ≤13.06,</td><td></td></tr>
</table>

4 years		Gross Motor ≤37.38,<br>Fine Motor 34.32,<br>Problem Solving ≤25.74,<br>Personal-Social ≤27.19.<br>Above cut-off = “low concern”<br><br>At 24 months “at-risk” cut-off:<br>Communication score ≤25.17,<br>Gross Motor ≤38.07,<br>Fine Motor 35.16,<br>Problem Solving ≤29.78,<br>Personal-Social ≤31.54.<br>Above cut-off = “low concern”<br><br>At 36 months “at-risk” cut-off:<br>Communication score ≤30.99,<br>Gross Motor ≤36.99,<br>Fine Motor 18.07,<br>Problem Solving ≤30.29,<br>Personal-Social ≤35.33.<br>Above cut-off = “low concern”<br><br>At 48 months “at-risk” cut-off:<br>Communication score ≤30.72,<br>Gross Motor ≤32.78,<br>Fine Motor ≤15.78,<br>Problem Solving ≤31.30,<br>Personal-Social ≤26.60.<br>Above cut-off = “low concern”	SECONDARY screen (for PEDS/LTSAE score) to determine “disability outcome” from weblink responses.<br><br>ASQ score = “at-risk” or QCHAT score “at-risk” referred to triage for follow up diagnostic screening at 2 years
Baseline outcome	Screen disability WMG-I group	A composite of ASQ-3 and Q-Chat positive screen result “at-risk”	SCREENING DISABILITY = Yes
	Screen disability Control group	GP confirms their “service as usual” scored the child “at-risk”	
	NO disability WMG-I group	A composite of ASQ-3 and Q-Chat positive screen result “low risk”	SCREENING DISABILITY = NO
	NO disability Control group	GP confirms their “service as usual” scored the child “low risk”	
2-year assessment	Mullen (MSEL)	T score of ≤30 (2 SD below the mean) indicate scores <3 percentile rank = “high risk”<br>T score of ≤30 (2 SD below the mean) indicate scores <10 percentile = at-risk<br>T score<br>of >30 indicate scores >10 percentile = low concerns	

		**If child scores <10 percentile = check Vineland score)*	Tests flagged "at-risk" when both Mullen and Vineland are scored below the 10th percentile.<br><br>ADOS flagged "at-risk" when Autism risk is scored positive.<br><br>Triage Team reviews and confirms whether "at-risk" or "low/no concern" pathway result from 2-year old diagnostic assessment.
	Vineland-3	Domain and ABC standard scores that fall in the range:<br>• 20-70 "low range" (reflect <3rd percentile)= "at-risk"<br>• 71-85 "moderately low" = (reflect 3-16th percentile).<br>• >10 percentile = low concerns<br>**If child scores <10 percentile = check Mullen score)*	
	ADOS-2 toddler	Overall total for <u>children with no words</u> is scored:<br>• 10-13 "mild-to-moderate concern" = "at-risk"<br>• 14+ "moderate to severe concern" = "at-risk"<br>• Scoring ≤9 = "low risk"<br><br>Overall total for <u>children with some words</u> is scored:<br>• 12+ "moderate to severe concern" = "at-risk"<br>• Scoring ≤11 = "low risk"	
2 year outcome	Diagnostic Disability in the WMG-I group and control group (all "at-risk" children +10% of "no-risk" children will be assessed)	A composite of ANY Positive "at-risk" score on the MSEL and VINELAND-3<br>OR<br>Positive "at-risk" score on the ADOS-2	SCREENING DISABILITY = Yes
		"Low concern" score on Mullen, Vineland and ADOS-2 toddler assessment	SCREENING DISABILITY = LOW RISK
