# Supplementary material for: Can we do better in identifying child developmental conditions early? Sensitivity and specificity of the Watch Me Grow Integrated (WMG-I) digital developmental screening and surveillance tool in Australia
Source: BMJ Open. 2026 Sep 25;16(9):e117341. doi: 10.1136/bmjopen-2026-117341 (PMC13629963; doi:10.1136/bmjopen-2026-117341)
Supplement: Supplementary file 2 [file bmjopen-2026-117341.reviewer_comments.pdf]

PEER REVIEW HISTORY

BMJ Open publishes all reviews undertaken for accepted manuscripts. Reviewers are asked to complete a checklist review form and are provided with free text boxes to elaborate on their assessment. These free text comments are reproduced below.

ARTICLE DETAILS

Title (Provisional)

Can we do better in identifying child developmental conditions early? Sensitivity and specificity of the Watch Me Grow Integrated (WMG-I) digital developmental screening and surveillance tool in Australia

Authors

Lam-Cassettari, Christa; Short, Kate; Garg, Pankaj; Descallar, Joseph; Khan, Feroza; Bradford, Catherine; Andersen, Isabella; Jalaludin, Bin; Liaw, Siaw-Teng; Woolfenden, Susan; Chambers, Aaron; Pritchard, Margo; Lingam, Raghu; Lawson, K D; Page, Andrew; Kohlhoff, Jane; Marron, Catherine; Heussler, Helen; Newcomb, Dana; de Mello, Nguyet; Murphy, Elisabeth; Colditz, Paul B; Kundukulam, Micheal; Samaranayake, Dimuthu; Raman, Shanti; Southcombe, Faye; Watch Me Grow Study Team, The; Scott, James; Eapen, Valsamma

---

VERSION 1 - REVIEW

**Reviewer**	**1**
**Name**	**Poulton, Alison**
**Affiliation**	**University of Sydney, Sydney Medical School Nepean**
**Date**	**11-May-2026**
**COI**	**I co-authored a paper submitted to Medicine Today with Valsamma Eapen.**

In this study the authors compared a digital tool (WMG-I), which, when ‘at risk’ results were obtained, was combined with LTSAE/PEDS+ASQ-3 to increase precision. The results are variously analysed using the WMG-I alone or using it when combined with the LTSAE/PEDS+ASQ-3. This probably explains the tables 2 and 3 having different numbers. But the differences between the WMG-I ‘at risk’ and the ‘index test positive’ need to be clearly defined in the body of the paper and in the legends of all the relevant tables.

I recommend a flow chart adding a second level for the WMG-I that indicates when the LTSAE/PEDS+ASQ-3 did and did not agree with the result from the WMG-I (or add this to the existing flow chart). Perhaps if you could construct a flow chart that shows how the 3 groups

described on p9 L 41-48 derive from the WMG-I screening and flow into the two groups on p10 L 25-28, then the differences between numbers might become clearer.

The authors rightly point out the importance of early identification of neurodevelopmental disorders as a pre-requisite for commencing early intervention. However, the fact that only 55% and 67% of those with significant concerns on screening with the WMG-I and the SaU respectively presented for further diagnostic testing/confirmation, suggests that this is an area that also needs to be addressed, because screening by itself will achieve little.

Specific points

1. On p9 L51-54 it is stated: 'As illustrated in Figure 1, for the sample in each group, the percentages of children with various screening outcomes and reference-standard assessment outcomes were calculated to yield sensitivity and specificity of the WMG-I and the SaU approach.'

Figure 1 – does not give percentages for the 4 groups at time 1: these could usefully be added. Even so, the sensitivity and specificity cannot be calculated from the figure without giving the test findings at time 2.

2. I note that the totals who attended for the final assessments differ between Fig 1 and Table 3. And Tables 2 and 3. Why is this?

3. Table 2 appears to imply that ASD is a subset of developmental delay (DD). This is confusing! The way the different terms are being used should be clearly defined in the methods and I would suggest categorising ASD (meeting ADOS criteria) separately from developmental delay – such that the toddlers can have ASD without DD, DD without ASD, DD and ASD, or neither ASD nor DD.

4. In the discussion p12 l 24-29 it is stated that 'An ideal screening tool is one with a high sensitivity and reasonable specificity as was found in this study, as the specificity can be improved by a two-stage process'. Can the authors please calculate these with the one and 2 stage screening ie the WMG-I with and without the LTSAE/PEDS+ASQ-3?

5. Please can you calculate whether a negative WMG-I score is significantly associated with a positive ADOS: what is the p-value?

6. A major potential limitation for the future implementation of this research into clinical practice is the limited uptake of further assessment among those with concerns detected at screening. This issue needs to be addressed in the discussion, along with suggestions for ways that this can be improved, to encourage such families to engage in therapy.

**Reviewer**	**2**
**Name**	**Schonhaut, Luisa**
**Affiliation**	**Clinica Alemana de Santiago SA**
**Date**	**11-May-2026**

**COI**	**None**

This is an interesting manuscript that evaluates the psychometric properties of a program rather than an instrument—the "Watch Me Grow Integrated (WMG-I) digital developmental surveillance"—which I find both interesting and conceptually challenging I had to read the manuscript several times to fully understand it. The abstract is the weakest part of the manuscript.

There are certain aspects included in the conclusions and in the "Strengths and Limitations of the Study" section, which do not have a clear connection to what is mentioned in the abstract.

The abstract does not make it clear which instruments comprise the primary developmental surveillance tool of the Watch Me Grow Integrated (WMG-I) digital developmental surveillance group. In the "Strengths and Limitations of the Study" section, they imply that what they applied was a screening method, not a surveillance tool. I think it is important to clarify that this was done using the Two-Step model. It is also unclear what the routine assessment (Service as Usual: SaU) consists of, or who administers the secondary screeners (upon reading the full manuscript, it becomes clear that this was performed by an evaluator blinded to the results and the group to which the participants belonged).

Finally, in the conclusions and the manuscript's contribution, they mention "Leveraging childhood immunization (≈90% reach, including in low-income countries) or other routine primary care contacts…"—which is not mentioned or implied in the abstract and does not address the manuscript's objectives.

Outcomes: The criteria for a positive diagnostic outcome on the standard reference assessment are not clear (are they repeating themselves? Or are these different severity criteria? Or is it "or" and "and"?). It states: A positive diagnostic outcome for the standard reference assessment was determined when a child met diagnostic criteria on the i) ADOS, ii) scored below the 10th percentile on any domain of both the MSEL and Vineland-3, or iii) met diagnostic criteria on the ADOS and scored below the 10th percentile on any domain of both the MSEL and Vineland-3. (This is repeated in the Statistical Analysis section.)

Statistics: They do not report which statistics they used to compare the sensitivity and specificity of both groups.

Results: They repeat the gender distribution in the first and second paragraphs of the results. I suggest consolidating this.

Tables: There is an issue with the periods (used as decimal separators) in Table 3.

Review the formatting of the references; it appears to me that some are incomplete.

**Reviewer**	**3**
**Name**	**Kelson, Zoe**

**Affiliation**	**University of Exeter, Mathematics**
**Date**	**17-May-2026**
**COI**	**None**

The current study is part of a prospective longitudinal cluster-randomised controlled trial evaluating the digital ‘Watch Me Grow’ Integrated (WMG-I) program compared to routine Surveillance as Usual (SaU) 18. The specific aim of this sub-study was to test the diagnostic accuracy for identifying any developmental condition in WMG-I compared with SaU.

Reviewer comments:

"Specificity was 66% (95%CI: 52·4%-79·5%) and 57% (95%CI: 38·7%-76·7%) respectively. " [Abstract]

Can the respective p-value for this study finding please also be reported in the Abstract?

"The data in this article were collected as part of a prospective longitudinal cluster-randomised controlled trial to evaluate the digital ‘Watch Me Grow’ developmental screening and ongoing monitoring program for children from 18 to 48 months (the WMG-I study). The study had approval from South Western Sydney Local Health District Human Research Ethics Committee (2020/ETH01625), University of New South Wales (2020/ETH01625), and University of Queensland (2021/HE000667). Trial registration: ANZCTR-12621000680864."

The original study has been suitably registered.

Can the study protocol and SAP for the original RCT please be provided?

"Prospective sample of parents of 16-to-35-month-old children attending 40 General practices (randomised to WMG-I or SaU) in culturally diverse metropolitan New South Wales (NSW) and Queensland, Australia, participating in a longitudinal cluster Randomised Controlled Trial. " [Abstract]

and

"All parents were invited to participate in the WMG-I study when presenting with a child aged 16-35 months at participating general practices for immunisation or other healthcare needs. Study locations reflect areas of large healthcare service provision with populations > 1 million people, with approximately 40% of residents born overseas "

Can the authors please further assess whether the included cohort can be considered to be demographically and characteristically representative?

"all parents completed sociodemographic questions as part of the trial entry"

Can the authors please confirm if there was any missing data?

"Forty general practices were recruited and randomised to either implement the digital 'Watch Me Grow Integrated' (WMG-I) approach for developmental surveillance or to routine

care, Surveillance as Usual (SaU) (21) in South-West Sydney, New South Wales (NSW) and Brisbane, Queensland (QLD), with 20 practices randomised to the WMG-I group and 20 to the SaU group. "

Can clustering by practice please be adjusted for in the analytical design?

Can the authors please confirm if there were any stratification variables in the original random allocation?

"All children with identified concerns and 10% of randomly selected children without concerns received a reference standard assessment using the Mullen Scale of Early Learning, Autism Diagnostic Observation Schedule and Vineland Adaptive Behaviour Scale. The primary outcome was diagnostic accuracy of WMG-I compared to SAU on the reference standard assessment tools." [Abstract]

and

"Following this, all children who screened positive for any developmental needs from both WMG-I and SaU groups as well as a random sample of 10% who screened negative from the baseline cohort were invited to participate in a reference standard developmental assessment..."

and

"As illustrated in Figure 1, for the sample in each group, the percentages of children with various screening outcomes and reference-standard assessment outcomes were calculated to yield sensitivity and specificity of the WMG-I and the SaU approach. "

Can the authors please clarify why only 10% of those 'without concerns' were included in the analysis?

"The diagnostic outcome was based on the reference-standard assessments using three gold standard assessments: the Mullen Scale of Early Learning (MSEL) 25, Vineland Adaptive Behaviour Scales Third Edition (Vineland-3) 26, and the Autism Diagnostic Observation Schedule (ADOS; child verbal ability determined whether the Toddler Module, ADOS-1 and ADOS-2 was administered)...

A positive diagnostic outcome for the standard reference assessment was determined when a child met diagnostic criteria on the i) ADOS, ii) scored below the 10th percentile on any domain of both MSEL andVineland-3, or iii) met diagnostic criteria on ADOS and scored below the 10th percentile on any domain of both MSEL and Vineland-3."

Can the authors please comment on the subjectivity and variability of the scales used?

"The STARD reporting guideline was used to draft this manuscript."

Thanks to the authors for providing a copy of the associated checklist.

"Fisher’s exact test was used to analyse baseline socio-demographic characteristics between the two groups of parents and children that attended a standard reference assessment at

age 2 years. Sensitivity, specificity, and positive and negative likelihood ratios were calculated for the outcomes from the baseline screening assessments compared to whether or not the scores on the Gold Standard assessments (ADOS, MSEL and Vineland) indicated probable developmental disability or age-appropriate development. Probable developmental disability was defined as i) met diagnostic criteria on ADOS (a measure of Autism), or ii) scores on both MSEL plus Vineland were <10th percentile. Fisher's exact test was used to compare test characteristics between the groups"

Suitable statistical tests have been applied by the authors.

Can other performance measures, such as AUC and accuracy, please be statistically compared by the authors (e.g. applying DeLong test to analyse differences in AUC)?

Can the authors please clarify whether the diagnosis of 'probable developmental disability or age-appropriate development' was based on an 'and' or an 'or' between criteria i) and ii) here?

"The sociodemographic characteristics of the children who received the reference standard assessment are shown in Table 1."

and

"Significantly more male than female children attended the assessment in the SaU group (75%) compared to the WMG-I group (54.6%)."

Can the authors please conduct a post-hoc exploratory analysis adjusting for sex?

"Of the total of 665 children recruited at baseline there were 355 males (53%). At baseline screening, 94 out of 417 (23%) children in the WMG-I group were identified as having developmental concerns using the WMG-I weblink compared to 43 out of 248 (17%) identified by GPs in the SaU group (p = 0·1137). Eighty-one children from both groups who screened positive at baseline and 69 children (random sample of 10%) who screened negative participated in the reference standard assessment"

Can the sample size numbers please be clarified by the authors in the text?

For instance, where does 81 come from, as 94+43 = 137? How is 69 10% of 665-137=528?

Can all children and their screening outcomes please be assessed in the study, rather than taking samples which may skew and reduce the power of the analyses?

"Of the 655 children in the study at age 2-years, 150 children received reference standard assessment"

Can the authors please provide an assessment of sample size?

"Of the 150 children who completed the reference standard assessment, 52 had been identified as having probable developmental disability on the automated dual screening weblink in the WMG-I group and 29 had been identified as having probable developmental disability by their GP in the SAU group. Of those who met criteria in the WMG-I group, 2

(4%) met only the ADOS criteria, 13 (25%) met the composite criteria (<10th percentile on both MSEL+Vineland-3) and 26 (50%) met criteria on all assessments. Of those who met the criteria in the SaU group, 4 (14%) met only the ADOS criteria, 6 (21%) met the composite criteria (<10th percentile on both MSEL+Vineland-3) and 11 (38%) met criteria on all assessments. "

Similarly, can the authors please clarify the numbers and description here?

In particular, 2+13+26= 51, not 52? 4+6+11= 21, not 29?

"A positive Likelihood Ratio (LR) of 2·7, 95% CI [1·8, 4·03] and negative LR of 0·096, 95% CI [0·03, 0·29] was found for the WMG-I group, with a positive LR of 1·7, 95% CI [1·2, 2·7] and negative LR of 0·4, 95% CI [0·2, 0·9] for the SaU group. "

Can the authors please report all performance metrics and their comparisons in the Results text, along with the precise p-values for statistical assessment (whether significant or not)?

Can the authors please further interpret the study findings here (e.g. what does a positive LR mean)?

"Study participants were recruited from a group of general practitioners that were expressed interest in developmental screening and surveillance and agreed to participate in the study, which may limit the generalisability of the results " [Strengths and Limitations of this study]

and

"Our study evaluated the diagnostic accuracy of the WMG-I digital screening and surveillance tool compared to SaU in a group of GPs that agreed to participate in the study and they may be more motivated to conduct developmental checks. However, it is noteworthy that despite all GPs expressing interest in developmental screening and surveillance, the lower sensitivity shown by the SaU group (17% identified) compared to the WMG-I group (23% identified) may be a result of GPs in the SaU missing cases of probable developmental concern as they are not utilising standardised tools for routine developmental screening and surveillance as part of their usual practice. Indeed, the overall rate of cases identified as having probable developmental concerns by WMG-I participants aligns with typical rates of developmental concerns in the general population as indicated by the Australian Early Development Census (AEDC) 4 . It should also be noted that statistically fewer girls (25%) were identified by SAU GPs compared to the proportion of boys (75%) identified, indicating that GPs may be less likely to look for developmental problems in female toddlers. We argue that the WMG-I digital screening and surveillance tool will help GPs to systematically engage in developmental screening and indicate when to refer and follow up actively instead of taking a 'watch and wait' approach"

The authors provide a Discussion on the study limitations.

VERSION 1 - AUTHOR RESPONSE

Reviewer: 1   Dr. Alison Poulton, University of Sydney

1. In this study the authors compared a digital tool (WMG-I), which, when 'at risk' results were obtained, was combined with LTSAE/PEDS+ASQ-3 to increase precision. The results are variously analysed using the WMG-I alone or using it when combined with the LTSAE/PEDS+ASQ-3. This probably explains the tables 2 and 3 having different numbers. But the differences between the WMG-I 'at risk' and the 'index test positive' need to be clearly defined in the body of the paper and in the legends of all the relevant tables.
I recommend a flow chart adding a second level for the WMG-I that indicates when the LTSAE/PEDS+ASQ-3 did and did not agree with the result from the WMG-I (or add this to the existing flow chart). Perhaps if you could construct a flow chart that shows how the 3 groups described on p9 L 41-48 derive from the WMG-I screening and flow into the two groups on p10 L 25-28, then the differences between numbers might become clearer.

Response: Thank you for the suggestion. We have clarified the Figure 1 flow chart to show how the *3 groups described on p9 derive from the WMG-I screening and flow through the two groups on p10.*

Figure 1. Study cohort and patient flow through the study

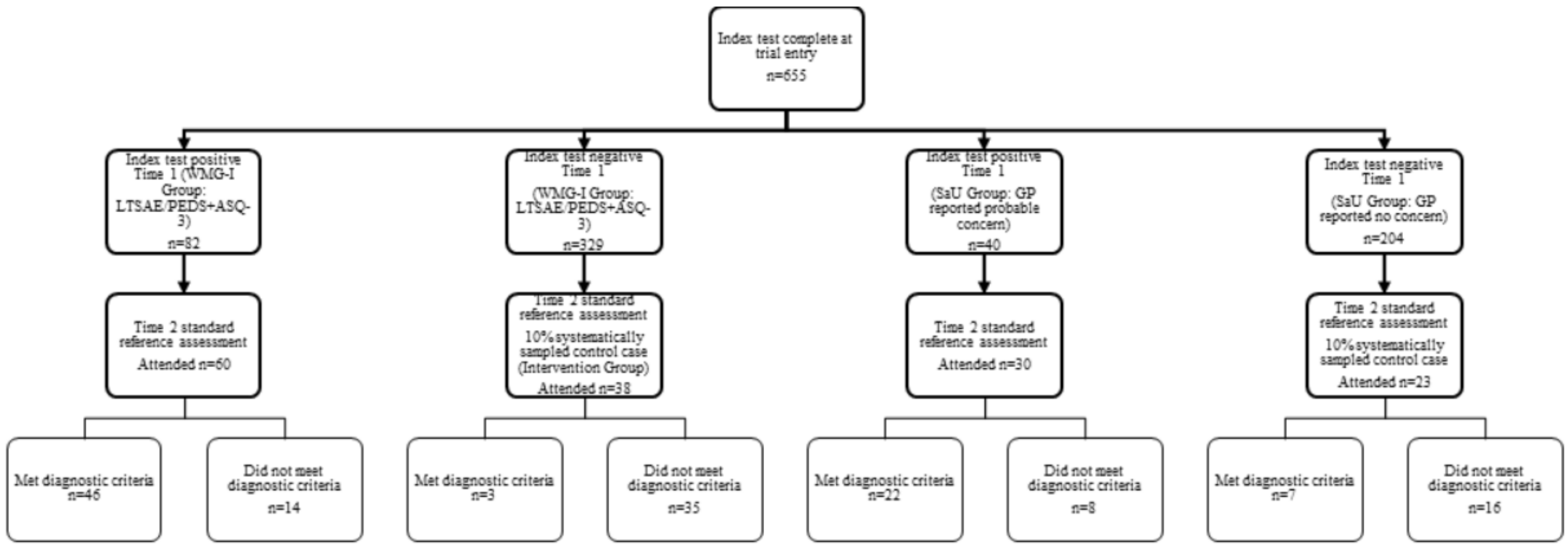

2. The authors rightly point out the importance of early identification of neurodevelopmental disorders as a pre-requisite for commencing early intervention. However, the fact that only 55% and 67% of those with significant concerns on screening with the WMG-I and the SaU respectively presented for further diagnostic testing/confirmation, suggests that this is an area that also needs to be addressed, because screening by itself will achieve little.

Response: Thank you for your constructive feedback. We have clarified the participant flow through the study in Fig 1 (see image above), which shows that 73% of WMG positive cases and 75% of the SaU positive cases attended the standard reference assessment. Reasons for non attendance at the standard reference assessment have also been included and in around half of the cases, the families that declined the gold standard reference assessment had indicated that they did not have any concerns to warrant a face-to-face assessment. Since this was a research study and hence we needed them to attend the gold-standard assessment even when they didn't have any

concerns, in clinical practice this will not be the case but will be based on the actual needs and hence we believe this will not be as much an issue.

We have also included further discussion of this in the limitations section "It should be noted that implementation of WMGI digital into clinical practice will require further 'buy-in' and upskilling of GPs/practice nurses in informing patients of the importance of developmental milestone checks and embed WMGI as part of their routine health care. It should also be noted that open and ongoing dialogue between GPs and parents will be needed in supporting uptake of further diagnostic testing.

Specific points:
1. On p9 L51-54 it is stated: 'As illustrated in Figure 1, for the sample in each group, the percentages of children with various screening outcomes and reference-standard assessment outcomes were calculated to yield sensitivity and specificity of the WMG-I and the SaU approach.'
Figure 1 – does not give percentages for the 4 groups at time 1: these could usefully be added. Even so, the sensitivity and specificity cannot be calculated from the figure without giving the test findings at time 2.

Response: This sentence has been revised for clarification and now reads "As illustrated in Figure 1, the patient flow through the various screening outcomes and reference-standard assessment outcomes were used to calculate the sensitivity and specificity of the WMG-I and the SaU approach"

2. I note that the totals who attended for the final assessments differ between Fig 1 and Table 3. And Tables 2 and 3. Why is this?

Response: Thank you for highlighting this typo. The total number of children that attended the final assessments in Fig 1, Table 2 and Table 3 have been revised.

3. Table 2 appears to imply that ASD is a subset of developmental delay (DD). This is confusing! The way the different terms are being used should be clearly defined in the methods and I would suggest categorising ASD (meeting ADOS criteria) separately from developmental delay – such that the toddlers can have ASD without DD, DD without ASD, DD and ASD, or neither ASD nor DD.

Response: Thank you, your suggestion has been implemented in the revised Table 2 and outcomes section in the revised manuscript on page 9.

4. In the discussion p12 l 24-29 it is stated that 'An ideal screening tool is one with a high sensitivity and reasonable specificity as was found in this study, ***as the specificity can be improved by a two-stage process'.*** *Can the authors please calculate these with the one and 2 stage screening ie the WMG-I with and without the LTSAE/PEDS+ASQ-3?*

Response: We have recalculated the sensitivity and specificity in the WMG-I group as below

Study arm	Baseline risk status	Gold Standard Assessment Diagnostic criteria met	Gold Standard Assessment Negative	Specificity (95% CI)	Sensitivity (95% CI)
WMG-I Group	Positive	48	20	98% (93.9%-100%)	59.2% (42.8%-75.6%)
	Negative	1	29		

5. Please can you calculate whether a negative WMG-I score is significantly associated with a positive ADOS: what is the p-value?

Response: We have calculated the association between WMG-I score and ADOS, with results below. A negative WMG-I score was associated with a negative ADOS score (p < 0.001).

WMG-I	ADOS = 1, n (%)	ADOS = 0, n (%)
Positive	32 (52.5)	29 (47.5)
Negative	2 (5.4)	35 (94.6)

p-value < 0.001
GEE with variance adjusted for small number of GP's

We have also added the following sentence to the end of the first paragraph in the results section on page 12: "A negative WMG-I score was associated with a negative ADOS score (p < 0.001)."

6. A major potential limitation for the future implementation of this research into clinical practice is the limited uptake of further assessment among those with concerns detected at screening. This issue needs to be addressed in the discussion, along with suggestions for ways that this can be improved, to encourage such families to engage in therapy.

Response: Thank you for this suggestion. As indicated above, this being a research study reference standard assessment was required even when children did not have any concerns, but we believe that in clinical practice as the follow up diagnostic assessment will be based on the clinical needs this will not be as much an issue. Further, we have expanded the limitations section to include the following point:

*"*It should be noted that implementation of WMGI digital into clinical practice will require further 'buy in' and upskilling of GPs/practice nurses in informing patients of the importance of developmental milestone checks and embed tools such as WMGI as part of their routine health care to aid in early identification and provision of appropriate supports if concerns are identified. *"*

Reviewer: 2 Dr. Luisa Schonhaut, Clinica Alemana de Santiago SA

Comments to the Author: This is an interesting manuscript that evaluates the psychometric properties of a program rather than an instrument—the "Watch Me Grow Integrated (WMG-I) digital developmental surveillance"—which I find both interesting and conceptually challenging I had to read the manuscript several times to fully understand it.
1. The abstract is the weakest part of the manuscript. There are certain aspects included in the conclusions and in the "Strengths and Limitations of the Study" section, which do not have a clear connection to what is mentioned in the abstract. The abstract does not make it clear which instruments comprise the primary developmental surveillance tool of the Watch Me Grow Integrated (WMG-I) digital developmental surveillance group. In the "Strengths and Limitations of the Study" section, they imply that what they applied was a screening method, not a surveillance tool. I think it is important to clarify that this was done using the Two-Step model.

Response: We apologize for the confusion. The abstract has been re-written to clarify that the WMGI uses a two-step model for the identification of developmental risk.

e.g., "Prospective sample of parents of 16-to-35-month-old children attending 40 General practices (randomised to WMG-I or SaU) in culturally diverse metropolitan New South Wales (NSW) and Queensland, Australia, participating in a longitudinal cluster Randomised Controlled Trial. Parents in the WMG-I group completed the WMG-I digital surveillance tool, which includes a primary screener and automated secondary screener (if concerns were reported). On completion, results and recommendations were transmitted to the parent and General Practitioner (GP) allowing discussion and further assessment. This was compared to SaU GP assessment. Once engaged, based on the date of birth of the child, the WMG-I sends automated reminders to take the check again at the next recommended ages, thereby allowing ongoing developmental monitoring/surveillance."

2. It is also unclear what the routine assessment (Service as Usual: SaU) consists of, or who administers the secondary screeners (upon reading the full manuscript, it becomes clear that this was performed by an evaluator blinded to the results and the group to which the participants belonged).

Response: The SaU GP's primarily reported clinical judgement of parental concern based on the routine checks while the reference standard assessment was conducted by an evaluator blinded to the results.

3. Finally, in the conclusions and the manuscript's contribution, they mention "Leveraging childhood immunization (≈90% reach, including in low-income countries) or other routine primary care contacts…"—which is not mentioned or implied in the abstract and does not address the manuscript's objectives.

Response: Thank you for highlighting this. We have added the opportunity to leverage primary care contacts such as immunization visits in the abstract and the sentence now reads: "Leveraging routine primary care contact such as immunization visits to

administer WMG-I digital, offers a scalable solution for increasing the uptake of developmental surveillance at a population level internationally. We have also included this unique opportunity for universal and equitable reach as a recommendation for future research. “Using tools such as WMGI alongside routine immunization programs, which have around 90% reach, will further enhance the equity and scalability of developmental screening and surveillance program internationally including in low and middle income countries.”

4. Outcomes: The criteria for a positive diagnostic outcome on the standard reference assessment are not clear (are they repeating themselves? Or are these different severity criteria? Or is it “or” and “and”?). It states: A positive diagnostic outcome for the standard reference assessment was determined when a child met diagnostic criteria on the i) ADOS, ii) scored below the 10th percentile on any domain of both the MSEL and Vineland-3, or iii) met diagnostic criteria on the ADOS and scored below the 10th percentile on any domain of both the MSEL and Vineland-3. (This is repeated in the Statistical Analysis section.)

Response: The “developmental disability” status was derived from known gold standard assessments. Autism risk was assessed using i) the ADOS (assessor trained to research reliability on this widely used semi-structured diagnostic assessment) *OR* ii) developmental disability was assessed through triangulation of the parent-reported Vineland-3 AND trained assessor administration of the MSEL. All measures require stringent training in their reliable execution (ADOS/MSEL), and outcomes were derived using the norm-based standardised scores to compare the child’s ability to peers of the same age/vocabulary level. Additionally, a clinical advisory group oversaw all assessment interpretations in case review meetings.

Thank you for this suggestion. The text describing how developmental risk is determined has been revised at the end of the Procedures section on page 9, as well as in the Outcomes section on page 10.

5. Statistics: They do not report which statistics they used to compare the sensitivity and specificity of both groups.

Response: When comparing sensitivity and specificity between the groups, we have calculated p-values from a Generalised estimating equation model to account for clustering by GP. An additional variance adjustment was performed to account for the fact there is a small (<40) GPs included in this analysis.

We have added the following text to the Methods – Statistical Analysis sub section (p.10)

“Generalised estimating equation with logit link and binomial distribution was used to compare the sensitivity and specificity between the groups, clustering by GP. The variance was adjusted to account for the small (<40) GPs, that were included as part of this analysis.”

6. Results: They repeat the gender distribution in the first and second paragraphs of the results. I suggest consolidating this.

Response: Thank you, the duplication has been removed. It now reads “Significantly more male than female children attended the assessment in the SaU group (75%) compared to the WMG-I group (54.6%). Of the total of 665 children recruited at baseline there were no other significant differences between sociodemographic variables for the WMG-I and the SaU groups.”

7. Tables: There is an issue with the periods (used as decimal separators) in Table 3.

Response: Thank you. The periods have been edited in Table 3 so they are formatted consistently throughout the Table.

8. Review the formatting of the references; it appears to me that some are incomplete.

Response: The references have been revised to ensure completeness.

Reviewer: 3 Prof. Zoe Kelson, University of Exeter
Comments to the Author:
The current study is part of a prospective longitudinal cluster-randomised controlled trial evaluating the digital ‘Watch Me Grow’ Integrated (WMG-I) program compared to routine Surveillance as Usual (SaU) 18. The specific aim of this sub-study was to test the diagnostic accuracy for identifying any developmental condition in WMG-I compared with SaU.

Reviewer comments:
1. "Specificity was 66% (95%CI: 52·4%-79·5%) and 57% (95%CI: 38·7%-76·7%) respectively. " [Abstract]
Can the respective p-value for this study finding please also be reported in the Abstract?
Response: We have now added the p-value for this comparison.
“There was a statistically significant difference with 93.9% sensitivity in the WMG-I group (95%CI: 88·1%-99.7%), and 75.9% (95%CI: 57.2%-94.6%) in the SaU group (p = 0·035); Specificity was 71.4% (95%CI: 54.7%-88.1%) and 66.7% (95%CI: 44.9%-88.4%) (p = 0.743),”
2. "The data in this article were collected as part of a prospective longitudinal cluster-randomised controlled trial to evaluate the digital ‘Watch Me Grow’ developmental screening and ongoing monitoring program for children from 18 to 48 months (the WMG-I study). The study had approval from South Western Sydney Local Health District Human Research Ethics Committee (2020/ETH01625), University of New South Wales (2020/ETH01625), and University of Queensland (2021/HE000667). Trial registration: ANZCTR-12621000680864."

The original study has been suitably registered.

Can the study protocol and SAP for the original RCT please be provided?

Response: The citation for the study protocol has been highlighted in the study design section on page 7, see "(the WMG-I study, for further information see the study protocol [18])."

3. "Prospective sample of parents of 16-to-35-month-old children attending 40 General practices (randomised to WMG-I or SaU) in culturally diverse metropolitan New South Wales (NSW) and Queensland, Australia, participating in a longitudinal cluster Randomised Controlled Trial. " [Abstract]

And "All parents were invited to participate in the WMG-I study when presenting with a child aged 16-35 months at participating general practices for immunisation or other healthcare needs. Study locations reflect areas of large healthcare service provision with populations > 1 million people, with approximately 40% of residents born overseas "

Can the authors please further assess whether the included cohort can be considered to be demographically and characteristically representative?

Response: The included cohort can be considered characteristically representative in comparison to sociodemographics collected as part of the 2021 Australian Census data. We have included the following text in the participants section "The sociodemographics including proportion of male births, average birthweight, speaking languages in addition to English at home, and holding a post school qualification aligns with the proportions shown in the Australian census data for South Western Sydney and Brisbane metropolitan areas (e.g., ABS, 2021)."

4. "all parents completed sociodemographic questions as part of the trial entry"
Can the authors please confirm if there was any missing data?

Response: There was no missing data

5. "Forty general practices were recruited and randomised to either implement the digital 'Watch Me Grow Integrated' (WMG-I) approach for developmental surveillance or to routine care, Surveillance as Usual (SaU) (21) in South-West Sydney, New South Wales (NSW) and Brisbane, Queensland (QLD), with 20 practices randomised to the WMG-I group and 20 to the SaU group. "
Can clustering by practice please be adjusted for in the analytical design?

Response: We have recalculated the confidence intervals for the sensitivities, specificities, positive LR and negative LR that appear in table 3, which adjusts for clustering by GP, with a variance inflation factor (Genders et al.). Under statistical analysis we have added the following sentence:

"Confidence intervals for these statistics were calculated with a variance inflation factor to account for clustering by GP."

When comparing sensitivities and specificities, the p-values have now been calculated from a Generalized Estimating Equation model with a logit link and binary distribution, with a further adjustment to the variance to incorporate the small number (<40) of GPs that are included in this analysis. Under statistical analysis, we have added the following:

"Generalised estimating equation with logit link and binomial distribution was used to compare the sensitivity and specificity between the groups, clustering by GP. The variance was adjusted to account for the small (<40) GPs, that were included as part of this analysis."

6. Can the authors please confirm if there were any stratification variables in the original random allocation?

Response: As per the study protocol randomisation was "conducted using minimisation across two factors, state and GP size by an independent statistician in the statistical software R using the 'Minirand' package. ". This has been clarified in the Randomisation and masking section on page 7.

7. "All children with identified concerns and 10% of randomly selected children without concerns received a reference standard assessment using the Mullen Scale of Early Learning, Autism Diagnostic Observation Schedule and Vineland Adaptive Behaviour Scale. The primary outcome was diagnostic accuracy of WMG-I compared to SAU on the reference standard assessment tools." [Abstract]
and
"Following this, all children who screened positive for any developmental needs from both WMG-I and SaU groups as well as a random sample of 10% who screened negative from the baseline cohort were invited to participate in a reference standard developmental assessment..."
and
"As illustrated in Figure 1, for the sample in each group, the percentages of children with various screening outcomes and reference-standard assessment outcomes were calculated to yield sensitivity and specificity of the WMG-I and the SaU approach. "
Can the authors please clarify why only 10% of those 'without concerns' were included in the analysis?

Response: Study resources did not permit more than 10% of negative screens to be invited to attend a face to face standard reference assessment. Additional text directing readers to the study protocol and citation have been included in the Study Design section on page 7. In addition, the sampling strategy has been clarified on page 3 of the manuscript and now reads "All children with identified concerns and 10% of systematically sampled children without concerns received a reference standard assessment".

8. "The diagnostic outcome was based on the reference-standard assessments using

three gold standard assessments: the Mullen Scale of Early Learning (MSEL) 25, Vineland Adaptive Behaviour Scales Third Edition (Vineland-3) 26, and the Autism Diagnostic Observation Schedule (ADOS; child verbal ability determined whether the Toddler Module, ADOS-1 and ADOS-2 was administered)...
A positive diagnostic outcome for the standard reference assessment was determined when a child met diagnostic criteria on the i) ADOS, ii) scored below the 10th percentile on any domain of both MSEL and Vineland-3, or iii) met diagnostic criteria on ADOS and scored below the 10th percentile on any domain of both MSEL and Vineland-3."
Can the authors please comment on the subjectivity and variability of the scales used?

Response: The "developmental disability" status was derived from known gold standard assessments. Autism risk was assessed using i) the ADOS (assessor trained to research reliability on this widely used semi-structured diagnostic assessment) *OR* ii) developmental disability was assessed through triangulation of the parent-reported Vineland-3 AND trained assessor administration of the MSEL. All measures require stringent training in their reliable execution (ADOS/MSEL), and outcomes were derived using the norm-based standardised scores to compare the child's ability to peers of the same age/vocabulary level.

A statement referring to the subjectivity of administration and determination of developmental risk has been included at the end of the Procedures section on page 9, as well as in the Outcomes section on page 10.

9. "The STARD reporting guideline was used to draft this manuscript."
Thanks to the authors for providing a copy of the associated checklist.

Response: Thank you for your feedback.

10. "Fisher's exact test was used to analyse baseline socio-demographic characteristics between the two groups of parents and children that attended a standard reference assessment at age 2 years. Sensitivity, specificity, and positive and negative likelihood ratios were calculated for the outcomes from the baseline screening assessments compared to whether or not the scores on the Gold Standard assessments (ADOS, MSEL and Vineland) indicated probable developmental disability or age-appropriate development. Probable developmental disability was defined as i) met diagnostic criteria on ADOS (a measure of Autism), or ii) scores on both MSEL plus Vineland were <10th percentile. Fisher's exact test was used to compare test characteristics between the groups"
Suitable statistical tests have been applied by the authors.

Response: Thank you for your encouraging feedback.

11. Can other performance measures, such as AUC and accuracy, please be statistically compared by the authors (e.g. applying DeLong test to analyse differences in AUC)?

Response: We have calculated the AUC for the WMG-I and SaU groups and compared between them. The following has been added to the Methods – Statistical analysis:

"AUC were calculated for the WMG-I and SaU groups, and compared with the Cluster-Robust Obuchowsli method to account for clustering by GP."

Under results, the following has been added:

"The AUC for WMG-I group was 0.827, and for SaU group was 0.713, and was not statistically significant different (p = 0.151). The accuracy was 82.7% in the WMG-I group, and 71.7% in SaU group."

12. Can the authors please clarify whether the diagnosis of 'probable developmental disability or age-appropriate development' was based on an 'and' or an 'or' between criteria i) and ii) here?

Response: Thank you for highlighting this oversight. The diagnosis of probable developmental disability was based on an "OR" between criteria. This has been clarified in the manuscript at the end of the Procedures section on page 9, as well as in the Outcomes section on page 10.

13. "The sociodemographic characteristics of the children who received the reference standard assessment are shown in Table 1."
and
"Significantly more male than female children attended the assessment in the SaU group (75%) compared to the WMG-I group (54.6%)."
Can the authors please conduct a post-hoc exploratory analysis adjusting for sex?

Response: When adjusting for sex, the p-values for differences in sensitivy was 0.0606, the p-values for differences in specificities was 0.8164.

The following statements have been added to the results section:

"Given the higher proportion of male children than females in the SaU compared with the WMG-I group, a post-hoc exploratory analysis was conducted which showed that after accounting for sex, the difference in sensitivities was not significant (p = 0.061)."

"The 4.7% difference in specificities was not significantly different (p = 0.743) and remained non-significant after adjusting for sex (p = 0.816)."

14. "Of the total of 665 children recruited at baseline there were 355 males (53%). At baseline screening, 94 out of 417 (23%) children in the WMG-I group were identified as having developmental concerns using the WMG-I weblink compared to 43 out of 248 (17%) identified by GPs in the SaU group (p = 0·1137). Eighty-one children from both groups who screened positive at baseline and 69 children (random sample of 10%) who screened negative participated in the reference standard assessment"

Can the sample size numbers please be clarified by the authors in the text?
For instance, where does 81 come from, as 94+43 = 137? How is 69 10% of 665-137=528?

Response: Thank you for highlighting this, we note there was a mistake. The section has been re-written to clarify the patient flow alongside the update to Figure 1.

15. Can all children and their screening outcomes please be assessed in the study, rather than taking samples which may skew and reduce the power of the analyses?

Response: Due to limited resources in providing a face-to-face play standardised assessment battery to children aged 24+ months, a systematic sample of ~10% of the total baseline participants were invited for the standardised assessments with an assessor blind to their baseline outcome and group. Systematic sampling was used to minimise selection bias. This has been clarified in the procedures section on page 9 "as well as a systematic sample of 10% who screened negative from the baseline cohort (every 10th participant) were invited to participate in a reference standard developmental assessment when aged ≥ 24 months of age."

Of the participants that screened positive and did not attend a standard reference assessment: 12 declined the opportunity to attend an assessment due to "being too busy" and/or having "no concerns"; 6 already had specialist appointments/diagnostics in progress and did not want their child to receive any additional assessments; 11 did not respond to any contact attempts or messages; 3 has relocated interstate. It should also be noted that we also had 17 decline the opportunity to participate in the standard reference assessment for research purposes due to being "too busy to attend a face-to-face assessment" because they had "no concerns" when contacting every 10th participant that had screened negative in the baseline cohort.

16. "Of the 655 children in the study at age 2-years, 150 children received reference standard assessment"
Can the authors please provide an assessment of sample size?

Response: The revised manuscript includes the following statement regarding sample size in the Statistical Analysis section on page 10.
"Sample size was based on a convenience sample as determined by those who completed the reference standard assessment."

17. "Of the 150 children who completed the reference standard assessment, 52 had been identified as having probable developmental disability on the automated dual screening weblink in the WMG-I group and 29 had been identified as having probable developmental disability by their GP in the SAU group. Of those who met criteria in the WMG-I group, 2 (4%) met only the ADOS criteria, 13 (25%) met the composite criteria (<10th percentile on both MSEL+Vineland-3) and 26 (50%) met criteria on all assessments. Of those who met the criteria in the SaU group, 4 (14%) met only the ADOS criteria, 6 (21%) met the composite criteria (<10th percentile on both

MSEL+Vineland-3) and 11 (38%) met criteria on all assessments. "
Similarly, can the authors please clarify the numbers and description here?
In particular, 2+13+26= 51, not 52? 4+6+11= 21, not 29?

Response: Thank you for highlighting this area of confusion. The numbers and results text have been re-written, in addition to revising the table caption and column headings in Table 2 as suggested by Reviewer 1 to improve clarity.

Table 2 Categorisation of the standardised reference assessment outcomes for Autism (ASD), or Developmental Delay (DD), or ASD+DD, or neither ASD nor DD on the Mullen Scale of Early Learning, Vineland Adaptive Behaviour Scales Third Edition, and Autism Diagnostic Observation Schedule.

Risk status at baseline	ASD without DD<br><br>(ADOS diagnostic criteria met)	DD without ASD<br><br>(Both MSEL+Vineland-3 scored <10 percentile)	ASD + DD<br><br>(ADOS diagnostic criteria met + MSEL & Vineland-3 <10th percentile)	Does not meet criteria for ASD or DD
WMG-I weblink identified "at-risk" at baseline	n=1	n=14	n=27	n=18
WMG-I 10% negative at baseline case	n=1	n=1	n=0	n=36
SaU GP identified " at-risk" at baseline	n=0	n=6	n=12	n=12
SaU 10% negative at baseline	n=4	n=0	n=3	n=16

18. "A positive Likelihood Ratio (LR) of 2·7, 95% CI [1·8, 4·03] and negative LR of 0·096, 95% CI [0·03, 0·29] was found for the WMG-I group, with a positive LR of 1·7, 95% CI [1·2, 2·7] and negative LR of 0·4, 95% CI [0·2, 0·9] for the SaU group. "
Can the authors please report all performance metrics and their comparisons in the Results text, along with the precise p-values for statistical assessment (whether significant or not)?
Can the authors please further interpret the study findings here (e.g. what does a positive LR mean)?

Response: Thank you for this suggestion. We have included the following interpretation of the likelihood ratio
"This indicates for both groups, that identification by GP of probable development disability has a moderate increase on the probability of meeting the criteria. In the WMG-I group, no identification by GP of probable development disability has a slight decrease in probability of meeting the criteria, whereas in the SaU group this effect was moderate."

In addition to the results stated above, we have added the sensitivities, AUC and accuracy in the results paragraph.

19. "Study participants were recruited from a group of general practitioners that were expressed interest in developmental screening and surveillance and agreed to participate in the study, which may limit the generalisability of the results " [Strengths and Limitations of this study]
and
"Our study evaluated the diagnostic accuracy of the WMG-I digital screening and surveillance tool compared to SaU in a group of GPs that agreed to participate in the study and they may be more motivated to conduct developmental checks. However, it is noteworthy that despite all GPs expressing interest in developmental screening and surveillance, the lower sensitivity shown by the SaU group (17% identified) compared to the WMG-I group (23% identified) may be a result of GPs in the SaU missing cases of probable developmental concern as they are not utilising standardised tools for routine developmental screening and surveillance as part of their usual practice. Indeed, the overall rate of cases identified as having probable developmental concerns by WMG-I participants aligns with typical rates of developmental concerns in the general population as indicated by the Australian Early Development Census (AEDC) 4 . It should also be noted that statistically fewer girls (25%) were identified by SAU GPs compared to the proportion of boys (75%) identified, indicating that GPs may be less likely to look for developmental problems in female toddlers. We argue that the WMG-I digital screening and surveillance tool will help GPs to systematically engage in developmental screening and indicate when to refer and follow up actively instead of taking a 'watch and wait' approach"
The authors provide a Discussion on the study limitations.

Response: Thank you for your thoughtful review and feedback.

VERSION 2 - REVIEW

**Reviewer**	**1**
**Name**	**Poulton, Alison**
**Affiliation**	**University of Sydney, Sydney Medical School Nepean**
**Date**	**21-Jul-2026**
**COI**	

P8 'The general developmental surveillance tools that were used in the study were the same as recommended by the respective state health department' Does this refer to SaU?

Fig 1 – needs legend defining the diagnostic criteria. I would assume that this meant they had either ASD, DD or both. The tests at time 2 also need to be specified.

P 12 para 2 is confusing and needs to be re-written. Is 'identified as having probable developmental disability… in the WMG-I group' the same as 'met criteria in the WMG-I group' ? Presumably 'A negative WMG-I' means 'did not meet criteria on the WMG-1'? Could

you decide either to use the terminology ‘met criteria’ vs ‘did not meet criteria’ or else use ‘positive’ vs ‘negative’ – obviously defining which equates to the other term used: ‘probable developmental disability’.

Table 2 would benefit from adding the total group N to column 1. If you gave the numbers you would not need to put ‘WMG-I 10%’. Why is ‘case’ written in one of the cells in column 1? ‘WMG-I 10% negative at baseline case’. WMG-I 10% negative’ is confusing. Would it not be clearer to use the descriptor ‘WMG-I negative (10% selected)’? The table needs a legend clearly defining all terms and abbreviations.

This is still very confusing. What is the index screening? It would appear that this is the WMG-1. If – as is stated – that everyone had the WMG-1, why is one group the called the WMG-1 group and the other the SaU group? Surely it is the weblink to the further screening that is the differentiating factor.

P8: “All parents were invited to participate in the WMG-I study when presenting with a child aged 16-35 months at participating general practices for immunisation or other healthcare needs between 2023-2025”. This was followed by informed consent and demographics. Then the WMG-1 group completed the weblink. This appears to be for the particular state developmental surveillance tools. If these demonstrated concern they went to the Ages and Stages ASQ etc.

In the GP group (SaU) they completed the 4 items. Did the GP have access to the WMG-1 findings for their patients or were they blinded?

I have constructed a flow chart of what I think is the design of the study.

I find the flow chart provided insufficiently detailed as it does not define ‘index screening’ and combines 2 stages as one.

If I have got it wrong and that the GP group did not complete the WMG-1 at all, does it not follow that all of the controls must have come from the WMG-1 cohort?

**** The reviewer provided a marked copy with additional comments. Please contact the publisher for full details. ****

**Reviewer**	**2**
**Name**	**Schonhaut, Luisa**
**Affiliation**	**Clinica Alemana de Santiago SA**
**Date**	**07-Aug-2026**
**COI**	

I congratulate you on your excellent work; with the improvements made, the study is much clearer. It is undoubtedly a model to be implemented in other countries around the world.

A few minor comments:

Abstract: The abstract is disorganized; where “Objectives” should appear, the introduction is included instead. Where it says “Design,” the “Objectives” are listed. The conclusions should be derived from the presented results and address the objectives. I suggest removing “such as vaccination visits” (unless this is explained in the methodology section). Furthermore, it is not possible to extrapolate the results to an international level. This is even noted among the limitations of the study: “Study participants were selected from a group of primary care physicians who expressed interest in screening and developmental monitoring and agreed to participate in the study, which may limit the generalizability of the results.”

Regarding the sentence: “It should also be noted that statistically fewer girls (25%) were identified by SAU GPs compared to the proportion of boys (75%) identified, indicating that GPs may be less likely to look for developmental problems in female toddlers” it would be interesting to know whether the researchers found differences in detection rate when comparing boys and girls. (Table 1 shows the sociodemographic characteristics of the children who received the reference standard assessment; it indicates that there were more girls in the study group compared to the SaU group, but I understand that it does not necessarily compare the percentage of girls identified as being at risk for developmental delays. If you keep the sentence, it would be interesting to include a referenced comment; The rate of developmental disorders and autism spectrum disorder is expected to be higher among boys than among girls; also, studies indicate that there is a greater risk of underdiagnosis of autism in females.

It is interesting that the screening rate was similar between both groups at 20%; I think this warrants some comment in the discussion.

VERSION 2 - AUTHOR RESPONSE

Reviewer 1 Comments to the Author:

P8 ‘The general developmental surveillance tools that were used in the study were the same as recommended by the respective state health department’ Does this refer to SaU?

Response: Thank you for your query. The wording has been revised to:

“The general developmental surveillance tools that were used in the WMG-I weblink were the same as recommended by the respective state health department…” (p8).

“A parent/child attending a SaU group general practice received routine care by their GP including clinical judgement of any parent concern and/or administration of any screeners the GP deemed relevant to their service provision (e.g., LTSAE/PEDS/ASQ-3 are recommended by

the respective state health departments)” (p9).

3. Fig 1 – needs legend defining the diagnostic criteria. I would assume that this meant they had either ASD, DD or both. The tests at time 2 also need to be specified.

Response: The Figure 1 legend has been revised to:

“Figure 1. Study cohort and patient flow through the baseline test for the Intervention group (WMG-I weblink) and SaU group (GP log of any concern) and Time 2 standard reference assessment (MSEL, VABS, ADOS). Time 2 outcome of “met diagnostic criteria” reflects cases where ADOS diagnostic criteria were met, or scores were <10th percentile on both MSEL and VABS.”

A footnote detailing the abbreviations has also been included.

4. P 12 para 2 is confusing and needs to be re-written. Is ‘identified as having probable developmental disability… in the WMG-I group’ the same as ‘met criteria in the WMG-I group’? Presumably ‘A negative WMG-I’ means ‘did not meet criteria on the WMG-1’? Could you decide either to use the terminology ‘met criteria’ vs ‘did not meet criteria’ or else use ‘positive’ vs ‘negative’ – obviously defining which equates to the other term used: ‘probable developmental disability’.

Response: Thank you for raising this concern. This paragraph has been re-written to clarify these points and now reads as follows:

“At baseline screening, 82 out of 411 (20%) children in the WMG-I group were positive for probable developmental concerns on the WMG-I weblink compared to 40 out of 204 (19.6%) children reported by the SaU GPs as positive for probable developmental disability (p = 1). Of these 122 children with probable developmental disability, 90 children and a further 61 children (the systematic sample of 10%) who screened negative participated in the reference standard assessments.

Of the 151 children who completed the reference standard assessments, 60 were positive on the automated dual screening weblink in the WMG-I group and 30 had been identified as positive for having probable developmental disability by their GP in the SAU group.”

5. Table 2 would benefit from adding the total group N to column 1. If you gave the numbers you would not need to put ‘WMG-I 10%’. Why is ‘case’ written in one of the cells in column 1? ‘WMG-I 10% negative at baseline case’. WMG-I 10% negative’ is confusing. Would it not be clearer to use the descriptor ‘WMG-I negative (10% selected)’? The table needs a legend clearly defining all terms and abbreviations.

Response: Thank you for these suggestions. The following changes have been made to Table 2:

- The total group N is now included in column 1.
- We have removed the word ‘case’ from cell 3 in column 1.
- We have used the descriptor ‘WMG-I negative (10% selected)’.
- A figure legend is included to define the terms that were not already defined in the Figure caption.

6. This is still very confusing. What is the index screening? It would appear that this is the WMG-1. If – as is stated – that everyone had the WMG-1, why is one group the called the WMG-1 group and the other the SaU group? Surely it is the weblink to the further screening that is the differentiating factor.

Response: We hope that the revisions in response to your queries above have now clarified that i) the WMG-I group completed their baseline test via the WMG-I weblink which contains the

screeners recommended by the state health department and ii) while the state government recommendation is that all children complete the developmental checks using the same questionnaires as used in WMG-I (LTSAE followed by ASQ-3 in NSW and PEDS followed by ASQ-3 in Queensland), often in practice, GPs who are funded by the Commonwealth Government are not obliged to conduct the checks or use these questionnaires recommended by the state government and hence the SaU group completed their baseline test based on their GPs provision of service as usual which is often the clinical judgement (may include any of the state recommended screens but this was not a requirement) as recorded in the SaU GPs log of 'concern' or 'no concern'.

7. P8: "All parents were invited to participate in the WMG-I study when presenting with a child aged 16-35 months at participating general practices for immunisation or other healthcare needs between 2023-2025". This was followed by informed consent and demographics. Then the WMG-1 group completed the weblink. This appears to be for the particular state developmental surveillance tools. If these demonstrated concern they went to the Ages and Stages ASQ etc.
In the GP group (SaU) they completed the 4 items. Did the GP have access to the WMG-1 findings for their patients or were they blinded?
I have constructed a flow chart of what I think is the design of the study.
I find the flow chart provided insufficiently detailed as it does not define 'index screening' and combines 2 stages as one.
If I have got it wrong and that the GP group did not complete the WMG-1 at all, does it not follow that all of the controls must have come from the WMG-1 cohort?
Response: Thank you for sketching a flow chart detailing which aspects would clarify the participant journey. The Figure 1 flow chart has been completely revised to illustrate the distinct pathways from trial entry for the two study groups through their baseline test, outcome from the baseline test, and pathways through the Time 2 standard reference assessment.

No 'screening results' were provided to SaU GPs as their patients did not complete the screeners on the WMG-I weblink. Once SaU families completed their consent and demographic information, SaU GPs provided their patient with 'service as usual' care which was often the clinical judgment (some may have completed the state recommended screening tools but this was not a requirement) as reported in the study log as to whether or not developmental concerns were present.

A systematic sample of 10% of children with "no concerns" on the WMG-I weblink were invited to attend the standard reference assessment for the intervention group, as well as a 10% systematic sample of children from the SaU group for which GP's reported "no concerns" to ensure that the standard reference test included children that had screened negative on the baseline test in both groups.

Reviewer. 2 Dr. Luisa Schonhaut, Clinica Alemana de Santiago SA
Comments to the Author:
I congratulate you on your excellent work; with the improvements made, the study is much clearer. It is undoubtedly a model to be implemented in other countries around the world.
Response: Thank you for your thoughtful feedback.

8. A few minor comments:
Abstract: The abstract is disorganized; where "Objectives" should appear, the introduction is included instead. Where it says "Design," the "Objectives" are listed. The conclusions should be

derived from the presented results and address the objectives. I suggest removing "such as vaccination visits" (unless this is explained in the methodology section). Furthermore, it is not possible to extrapolate the results to an international level. This is even noted among the limitations of the study: "Study participants were selected from a group of primary care physicians who expressed interest in screening and developmental monitoring and agreed to participate in the study, which may limit the generalizability of the results."

Response: Thank you for these suggestions. The abstract has been revised to clarify the objectives and the study design and now reads as follows:

"Objectives

While child developmental checks are universally recommended, engagement with developmental surveillance programs is low globally. To address this policy to practice gap, this study examined diagnostic accuracy of the "Watch Me Grow" integrated (WMG-I) digital developmental screening and ongoing surveillance approach.

Design

This sub-study examined diagnostic accuracy of the WMG-Integrated (WMG-I) digital approach compared to routine assessment (Service as Usual: SaU) in a prospective sample.

We have removed the words "such as vaccination visits".

9.Regarding the sentence: "It should also be noted that statistically fewer girls (25%) were identified by SAU GPs compared to the proportion of boys (75%) identified, indicating that GPs may be less likely to look for developmental problems in female toddlers" it would be interesting to know whether the researchers found differences in detection rate when comparing boys and girls. (Table 1 shows the sociodemographic characteristics of the children who received the reference standard assessment; it indicates that there were more girls in the study group compared to the SaU group, but I understand that it does not necessarily compare the percentage of girls identified as being at risk for developmental delays. If you keep the sentence, it would be interesting to include a referenced comment; The rate of developmental disorders and autism spectrum disorder is expected to be higher among boys than among girls; also, studies indicate that there is a greater risk of underdiagnosis of autism in females.

It is interesting that the screening rate was similar between both groups at 20%; I think this warrants some comment in the discussion.

Response: As suggested, a post hoc analysis was conducted to compare the detection rates in boys and girls. In the cohort, 66% of males and 57% of females were screened positive at time 1 and was not statistically different ($p = 0.3$). Of those with a reference standard positive result, 92% of males and 76% of females were screened positive at time 1, which was not significantly different ($p = 0.07$).

In the WMG-I group, 67% of males and 59% of females were screened positive at time 1 and was not statistically different ($p = 0.51$). Of those with a reference standard positive result, 97% of males and 89% of females were screened positive at baseline and were not significantly different ($p = 0.55$).

In the SaU group, 64% of males and 52% of females were screened positive at time 1 and was not statistically different ($p = 0.57$). Of those with a reference standard positive result, 86% of males and 43% of females were screened positive at baseline, and were significantly different ($p = 0.04$).

We have added the following sentence in the results
“Given that there were differences in the proportion of males and females in the SaU group, post-hoc analysis was conducted of children that were in the SaU group with a positive reference standard result and found that 86% of males (18/21) and 43% of females (3/7) were screened positive at time 1 ($p = 0.04$).”

In addition a statement and reference regarding evidence on sex differences in ASD has been added in the limitations section “This aligns with evidence for a male predominance in the diagnosis of ASD and greater difficulty in diagnosing females with ASD [39]. A recent population study demonstrated that diagnosis of ASD occurs later for females than males, although differences in the ratio of male to female diagnosis reduce as children reach 10 years of age [40].”
